# Identification of WRKY transcription factor family genes in *Pinus massoniana* Lamb. and their expression patterns and functions in response to drought stress

**DOI:** 10.1186/s12870-022-03802-7

**Published:** 2022-09-01

**Authors:** Shuang Sun, Hu Chen, Zhangqi Yang, Jingyu Lu, Dongshan Wu, Qunfeng Luo, Jie Jia, Jianhui Tan

**Affiliations:** 1 Key Laboratory of Central South Fast-Growing Timber Cultivation of Forestry Ministry of China, Guangxi Forestry Research Institute, Nanning, 530002 PR China; 2 Guangxi Key Laboratory of Superior Timber Trees Resource Cultivation, Nanning, 530002 PR China; 3 Engineering Technology Research Center of Masson Pine of National Forestry and Grassland Administration, Nanning, 530002 PR China

**Keywords:** *Pinus massoniana* Lamb., WRKY transcription factor, Drought stress, Exogenous hormone, Expression pattern, Functional study

## Abstract

**Background:**

*Pinus massoniana* Lamb. is the timber species with the widest distribution and the largest afforestation area in China, providing a large amount of timber, turpentine and ecological products. Seasonal drought caused by climate warming severely constrains the quality and growth of *P. massoniana* forests. WRKY transcription factors play an important role in plant responses to abiotic stress. In this study, the molecular mechanisms by which *P. massoniana* responds to drought stress were analysed based on the *P. massoniana* WRKY (PmWRKY) family of genes.

**Results:**

Forty-three PmWRKYs are divided into three major families, 7 sub-families, and the conserved motifs are essentially the same. Among these 43 *PmWRKYs* express under drought stress but with different expression patterns in response to stress. *PmWRKYs* respond to drought stress induced by exogenous hormones of SA, ABA, and MeJA. The expression of *PmWRKY6*, *PmWRKY10*, and *PmWRKY30* up-regulate in different families and tissues under drought stress, while *PmWRKY22* down-regulate. Transgenetic tobaccos of *PmWRKY31* are with lower malondialdehyde (MDA) content and higher proline (Pro) content than wild type (WT) tobaccos. In transgenic tobaccos of *PmWRKY31*, expression levels of related genes significantly improve, and drought tolerance enhance.

**Conclusions:**

This study analysed the molecular biological characteristics of PmWRKYs and investigated the expression patterns and functions of *PmWRKYs* in response to drought stress in *P. massoniana*. The results of this study provide a basis for in-depth research of the molecular functions of *PmWRKYs* in response to drought stress.

**Supplementary Information:**

The online version contains supplementary material available at 10.1186/s12870-022-03802-7.

## Background

Transcription factors (TFs) regulate gene expression by combining with specific *cis*-acting elements to form a unique regulatory network and play important roles in the abiotic stress response, growth and development of plants [1–3]. The WRKY TFs were first cloned from sweet potato [4]. Later, WRKY TFs were found in a variety of plants, including *Arabidopsis thaliana* [5], *Oryza sativa* [6] and *Populus trichocarpa* [7]. WRKYs have a highly conserved domain composed of 60 amino acids that contains a heptapeptide structure with a highly conserved WRKYGQK domain at its N-terminus, which is related to DNA binding activity [8]. Although the heptapeptide structure is highly conserved, WRKYGQK still has variations, such as WRKYGKK, WKKYGQK, and WRKYGRK [7]. In addition, gene function changes with the variation in conserved structure [9]. The C2H2 (CX_4-5_CX_22-23_HXH) and C2HC (CX_7_CX_23_HXC) zinc finger structures at the C-terminus participate in protein–protein interactions (PPIs) and assist in DNA binding [8, 10]. The WRKY genes can bind to the *cis*-acting element of the downstream gene promoter, which contains a very conserved (C/T)TGAC(T/C) sequence called the W-box [11]. WRKY genes bind to this element, thereby activating downstream gene expression. In addition, WRKY genes can also function by combining with elements such as W-box, PRE4, PRE2, G-box, WK box and SURE [12–14].

Drought is an important abiotic stress factor that hinders plant growth and development and is a frequent cause of damage. Plants can regulate their resistance to drought stress through the WRKY regulatory network and hormone signal transduction. For example, *HbWRKY1* expression in *Hevea brasiliensis* [15] and *HrWRKY21* expression in *Hippophae rhamnoides* [16] are upregulated in response to drought stress. *GhWRKY41* and *ThWRKY2* enhance the drought tolerance of cotton and *Tamarix hispida* [17, 18]. *EjWRKY17* from *Eriobotrya japonica* enhances drought tolerance in transgenic *Arabidopsis* [19]. WRKY genes can also improve the drought tolerance of plants by increasing osmotic regulatory substances, such as soluble sugar and proline (Pro) and other contents, and reducing the content of malondialdehyde (MDA) [20, 21].

*Pinus massoniana* Lamb., a major plantation timber species in southern China, has excellent characteristics, such as barren tolerance, strong adaptability, fast growth, and a wide range of applications [22, 23]. However, the uneven rainfall distribution and seasonal drought in southern China [24], severely restrict the growth of *P. massoniana*. Global warming and environmental deterioration further exacerbate this problem. Therefore, research on the drought tolerance of *P. massoniana* is of great importance. There are relatively few studies on the drought tolerance mechanisms of *P. massoniana* at the molecular level. Whole-genome sequencing of the *P. massoniana* genome has not yet been carried out due to its large size (expected size of approximately 24 Gb, gigabase pairs, chromosome-level assembly). Therefore, the identification of *P. massoniana* WRKY (PmWRKY) based on transcriptome analysis is the top choice. In this study, we identified the genes in the PmWRKY family in *P. massoniana* families with different drought tolerance based on transcriptome data and analysed their physicochemical properties, phylogenetic evolution, and conserved motifs. We also analysed the expression patterns of 4 *PmWRKYs* under hormone treatment and drought stress and the drought tolerance function of *PmWRKY31* to provide a reference for the further in-depth study of the functions of *PmWRKYs* and their mechanisms of response to drought stress.

## Results

### Identification and physiochemical characteristics of PmWRKYs

Based on previous full-length transcriptome data of the second and third generations, the gene sequences annotated WRKY were obtained. A total of 496 sequences were obtained using HMMER software and the BLAST method. After removing redundant sequences, 281 candidate sequences were obtained. The conserved WRKY domains of candidate protein sequences were analysed using NCBI CD search, SMART, and Pfam. After the sequences that contain incomplete WRKY domains or do not contain WRKY domains were removed, 43 *PmWRKYs* were finally identified and named based on homologous sequence data (Supplementary Table 1).

Analysis of the physicochemical properties and functional structure of proteins revealed that the PmWRKY proteins encode 120 (PmWRKY35) to 908 (PmWRKY1) amino acids; the molecular weights of the PmWRKY proteins range from 13.48 (PmWRKY35) to 98.15 (PmWRKY1) kDa. The theoretical isoelectric point (pI) values for PmWRKYs range from 4.68 (PmWRKY10) to 10.23 (PmWRKY40), with an average pI of 7.93. Among the PmWRKYs, 15 have a pI < 7, which is relatively acidic, and 28 have a pI > 7, which is alkaline. The instability coefficient of PmWRKY40 protein is 36.07, and the instability coefficients of 42 PmWRKY proteins are all greater than 40, indicating that they are unstable proteins. Among the 43 PmWRKY proteins, only PmWRKY25 (-0.459), PmWRKY37 (-0.320), and PmWRKY39 (-0.307) have an average hydropathicity > -0.5, indicating that they are hydrophilic; the rest are hydrophobic. The subcellular localization of all proteins was predicted to be in the nucleus, with no transmembrane structure (Supplementary Table 1).

### Analysis of the phylogenetic, conserved motifs and multiple sequence alignments of *PmWRKYs*

**Fig. 1:**
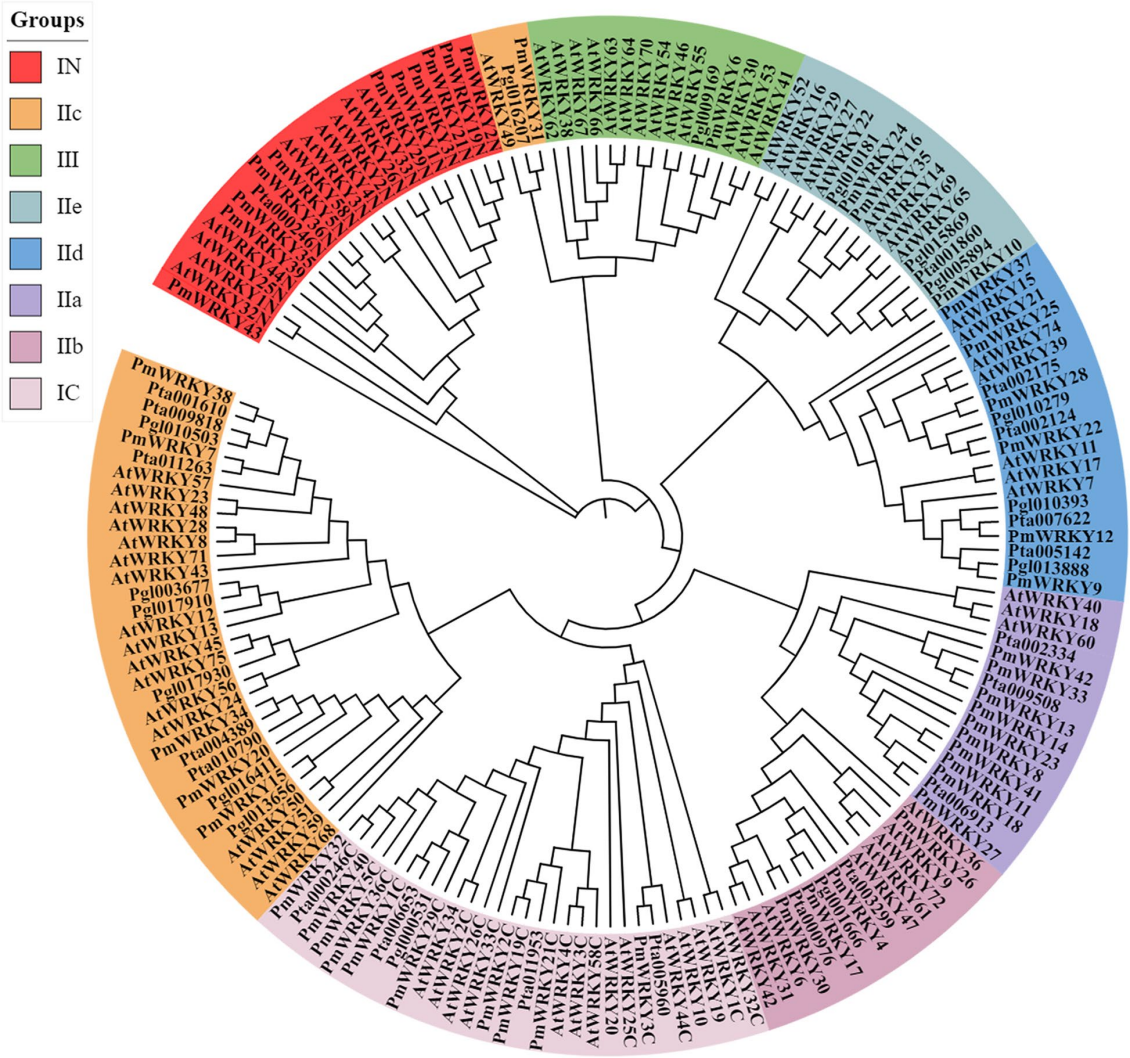
Phylogenetic tree analysis of PmWRKYs in *P. massoniana*. Reconstructed phylogenetic trees for *P. massoniana*., *Arabidopsis* (At, 72), *Pinus taeda* (Pta, 20), and *Picea glauca* (Pgl, 16). Different colours represent different subfamilies. The phylogenetic trees were generated using MEGA 7 software and iTOL

The phylogenetic tree showed that the WRKY family can be classified into families I, II and III. Family I, which contains 2 WRKY domains and a C2H2 zinc finger structure, is further categorized into the IC and IN subfamilies. Family II, which contains a WRKY domain and a C2H2 zinc finger structure, is categorized into subfamilies IIa, IIb, IIc, IId and IIe. Family III contains a WRKY domain and a C2HC zinc finger structure [25, 26]. Families I, II and III include 13 PmWRKYs, 29 PmWRKYs, and 1 PmWRKY (PmWRKY6), respectively. The IIa, IIb, IIc, IId and IIe subfamilies includes 10, 4, 6, 6, and 3 PmWRKYs, respectively. In addition, *P. massoniana* has a close genetic relationship with *Pinus taeda* and *Picea glauca* (both in the Gymnosperm Pinaceae) (Fig. 1).

**Fig. 2:**
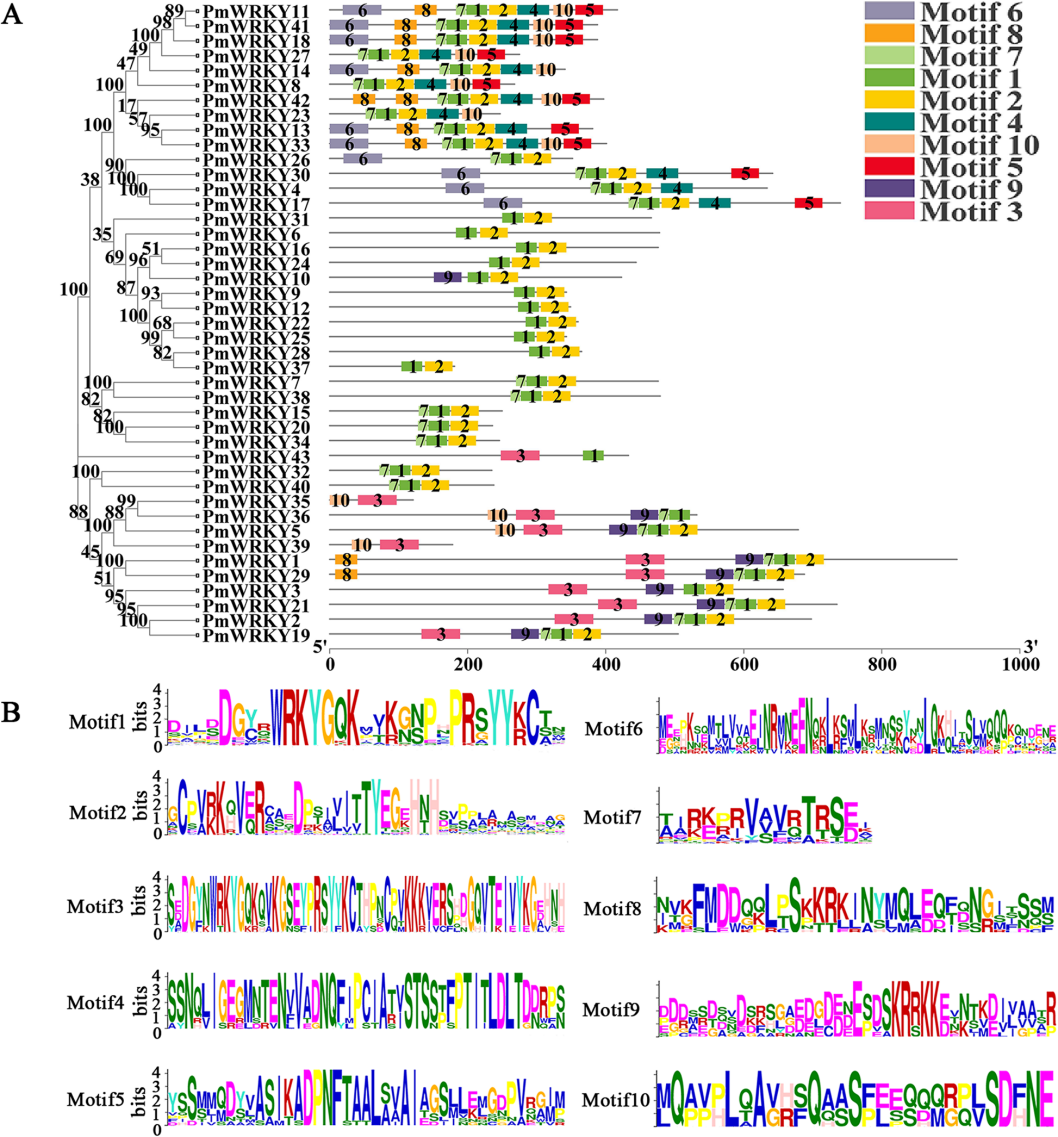
Analysis of the conserved motifs of PmWRKYs in *P. massoniana*. The 10 conserved motifs in a are represented by different numbers and colours, and the motifs in **b** correspond to those in **a**

Analyses of conserved gene motifs revealed 10 conserved motifs with a length of 15–57 amino acids. Motif1 and Motif3 are conserved heptapeptide domains of WRKY. Motif1 and Motif2 distribute throughout all subfamilies, while Motif3 only distributes in subfamily I. Motif4, Motif5, and Motif6 distribute in subfamilies IIa and IIb. Motif7 distributes in family I and subfamilies IIa, IIb and IIc. Motif8 and Motif10 distribute in family I and subfamily IIa. Motif9 distributes in family I and subfamily IIe. The evolution of WRKY genes is highly conserved, and they may have similar functions. However, there are also inconsistent conserved motifs contained in the same protein subfamily; for example, PmWRKY10 in subfamily IIe contains more Motif9 than does other members (Fig. 2a, b).

Multiple sequence alignment of PmWRKY protein sequences revealed that the protein sequences of the same subfamily genes have high similarity, further indicating that they may have similar gene functions. Most of the PmWRKY protein sequences contain the conserved heptapeptide WRKYGQK at the N-terminus, and only 5 proteins in subfamilies IN, IId, and IIc have amino acid variations, i.e., WTKYGKR, WRKYGQN, WRKYGKK, and WRKYGRK. There is a variable zinc finger structure at the C-terminus of the sequence, such as the deletion of the zinc finger structure in the C-terminus conserved domain of PmWRKY36 and the presence of zinc finger structure variation in PmWRKY39 and PmWRKY43 proteins (Supplementary Fig. 1).

### Protein–protein interactions of PmWRKYs

**Fig. 3:**
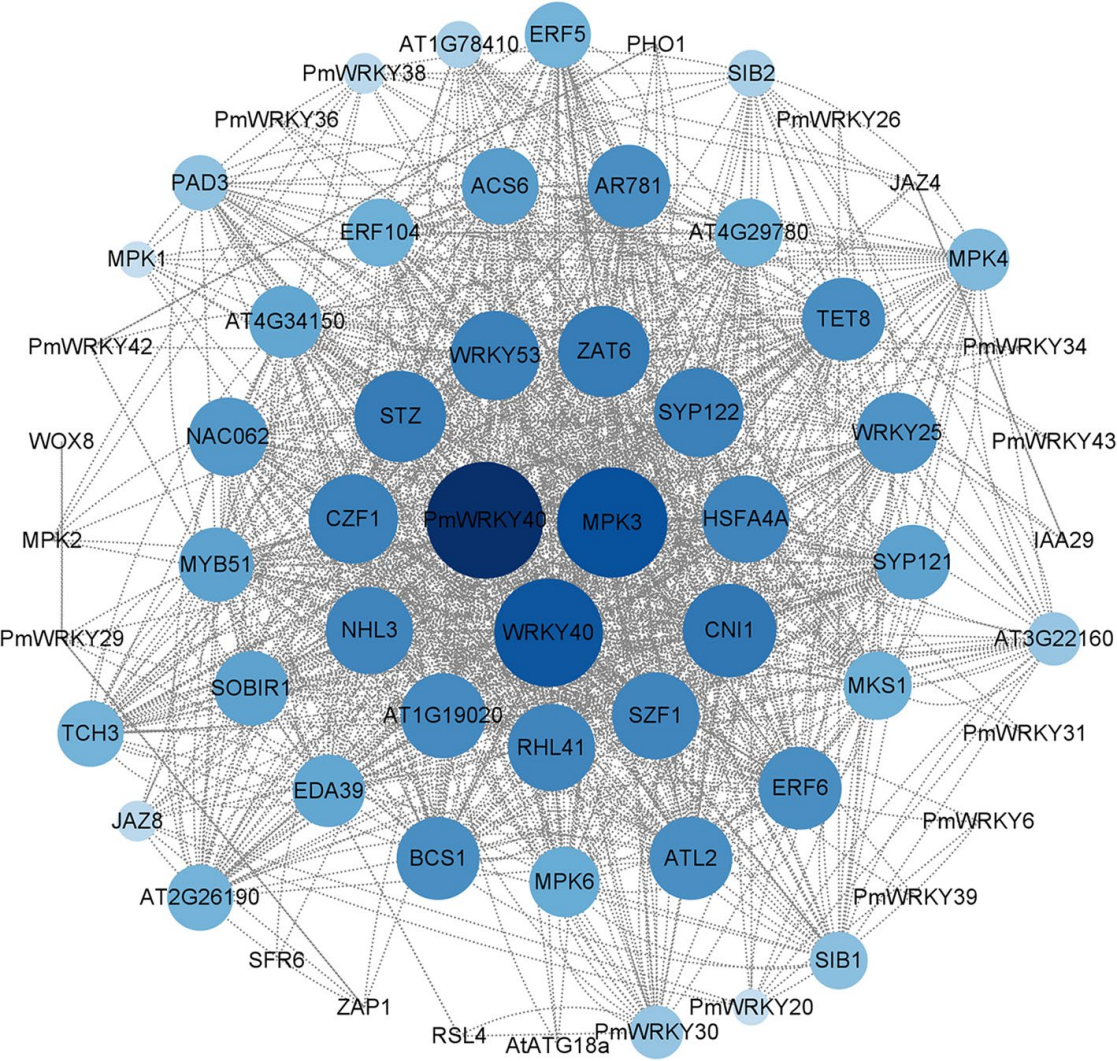
Prediction of the protein–protein interaction (PPI) network between PmWRKYs and *Arabidopsis* proteins. The PPI network of PmWRKYs was constructed using STRING and Cytoscape software and was analysed and predicted based on the WRKY proteins of *Arabidopsis*

The PPI network of PmWRKYs (Fig. 3) shows that most PmWRKYs can interact with more than 1 protein, e.g., PPIs between PmWRKY40 and PmWRKY30 and PPIs between PmWRKY6 and PmWRKY20. PmWRKY40 (AtWRKY33) plays an important role in the entire PPI network. AtWRKY33 *is* involved in the response to various abiotic stresses. Hence, PmWRKY40 may have similar functions [27].

### Expression analysis of *PmWRKY*s by RNA-seq

**Fig. 4:**
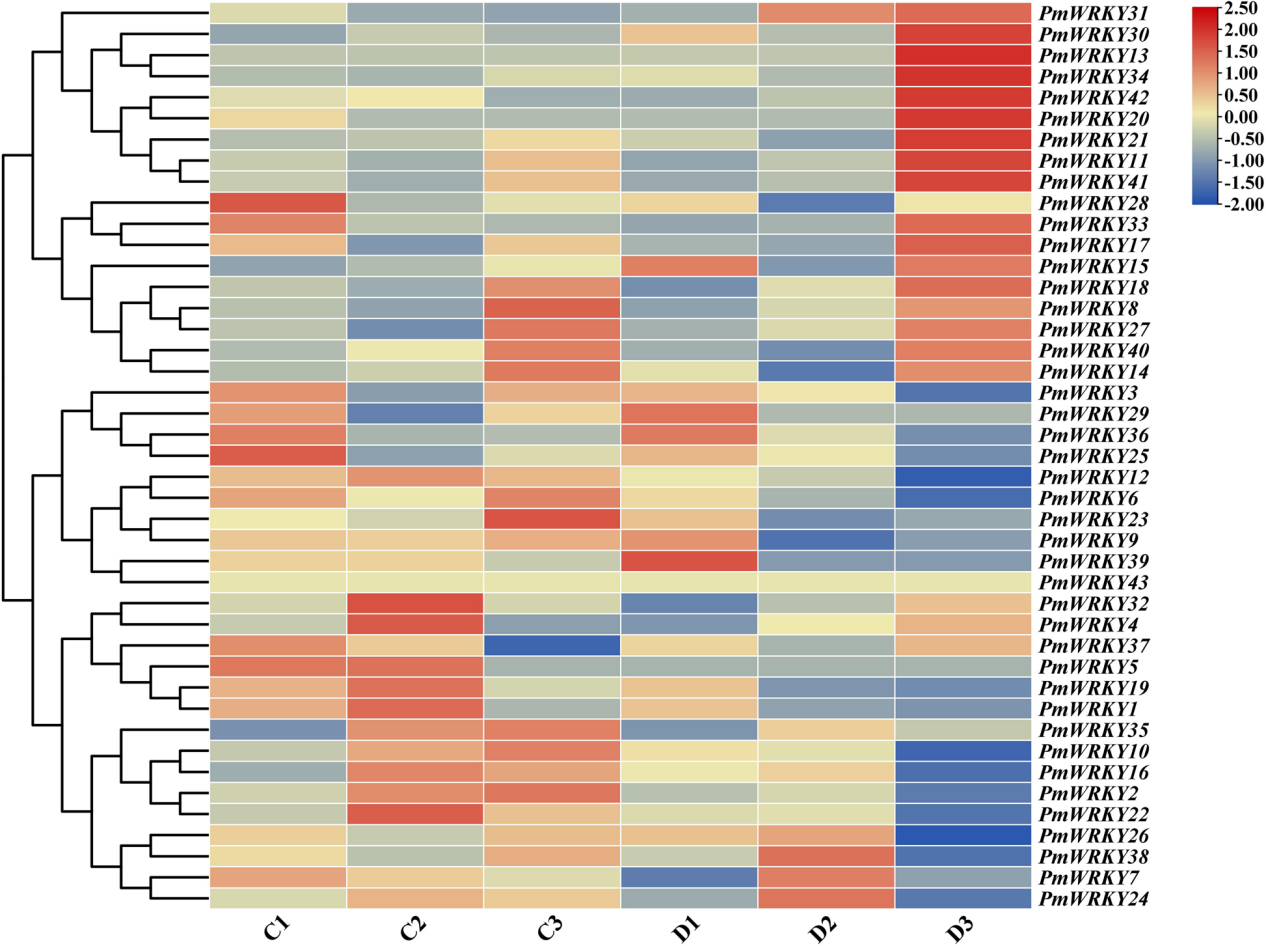
Heatmap of *PmWRKY* expression using RNA-seq data. Blue represents low expression levels, and red represents high expression levels. C and D represent the control group (normal growth, C) and the treatment group (drought stress, D), respectively; 1, 2, and 3 represent the control and drought treatments on the 7th day, 7 h after rewatering on the 8th day, and the 8th day, respectively. TBtools was used to generate the gene expression heatmap

An expression heatmap of 43 *PmWRKYs* under drought conditions was generated based on the transcriptome data (Fig. 4). All the genes were expressed during drought stress. The expression of 4 genes peaked on the 7th day of drought (D1), expression of 4 genes peaked on the 7th day of drought (D2), that of 15 genes peaked on the 8th day of drought (D3), and that of the remaining genes all peaked on C1-C3. With FDR ≤ 0.001 and |log2FC|≥ 2 as significant difference gene screening standards, at the same time combined with the phylogenetic tree, we eventually chose four differentially expressed genes, *PmWRKY6*, *PmWRKY10*, *PmWRKY22*, and *PmWRKY30*, for research of expression patterns under drought stress after treatments of hormones.

### Analysis of the expression of *PmWRKYs* under hormone treatments and drought stress

**Fig. 5:**
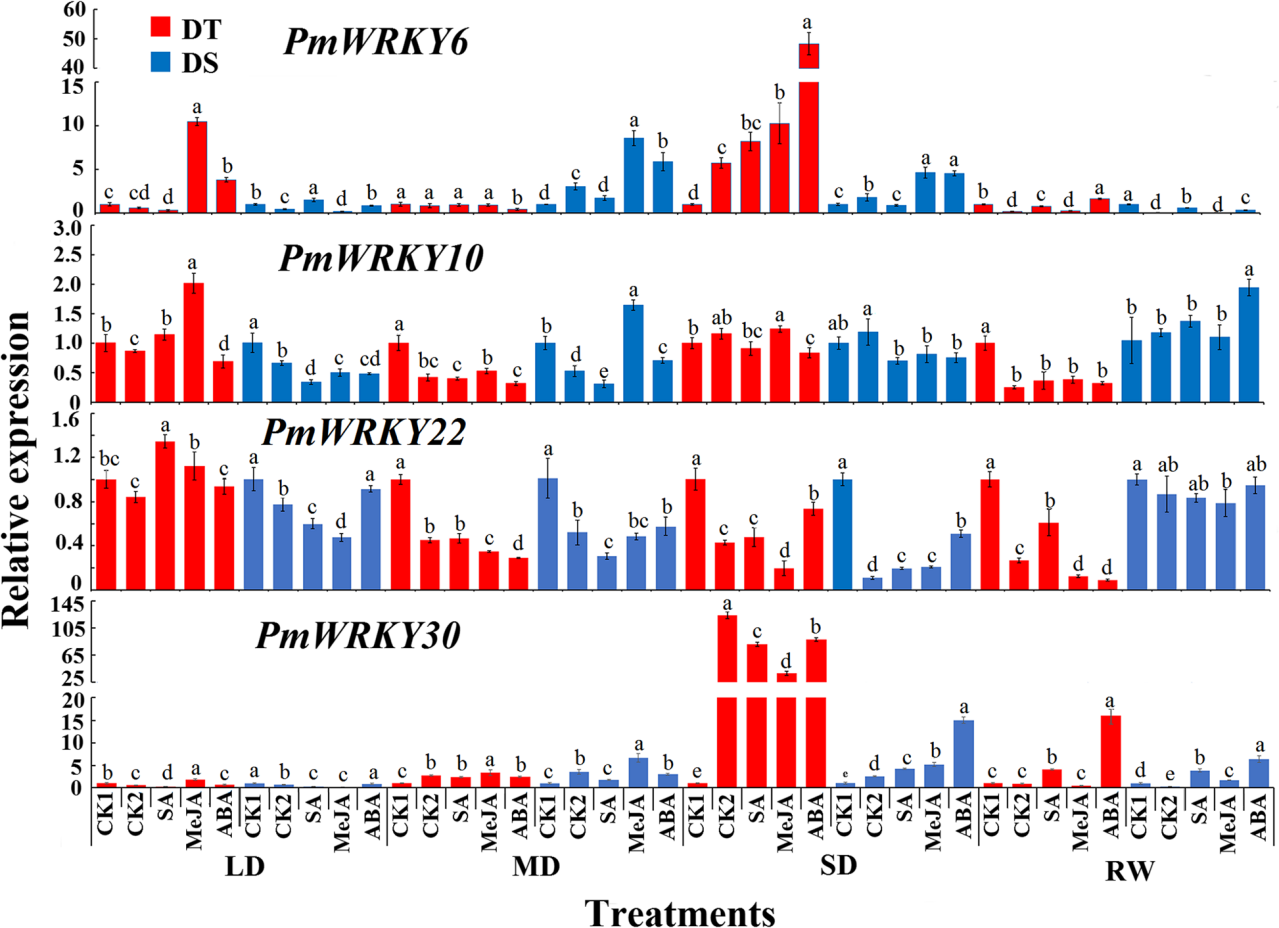
The expression patterns of *PmWRKYs* in leaves of the drought-tolerant and drought-sensitive family during drought stress. LD indicates light drought, MD indicates moderate drought, SD indicates severe drought, and RW indicates rewatering. DT indicates drought-tolerant, DS indicates drought-sensitive. Different lowercase letters indicate significant differences at *P* < 0.05

In needles, *PmWRKYs* had same expression pattern in different drought-resistant families, but also had different expression patterns. For example, expression of *PmWRKY6* was earlier induced by MeJA and ABA in DT families, while it was induced by MD stress in DS families. The expression of *PmWRKY6* was induced by SA, MeJA and ABA in DT family in response to drought stress. *PmWRKY10* and *PmWRKY22* showed higher expression levels in DT family than in DS family at different stress stages. However, after re-watering, the expression levels of PmWRKY10 and *PmWRKY22* in DS families were significantly higher than those in DT families. Combined with the phenotypic data, it was suggested that DS families need rapid up-regulation of these genes to cope with drought stress, while DT families showed more durable drought resistance. Under SD stress, the expression of *PmWRKY30* increased sharply in DT families, but its expression under hormone treatments was lower than that of CK2, while its expression of hormone treatments was higher than that of CK2 in DS families (Fig. 5).

**Fig. 6:**
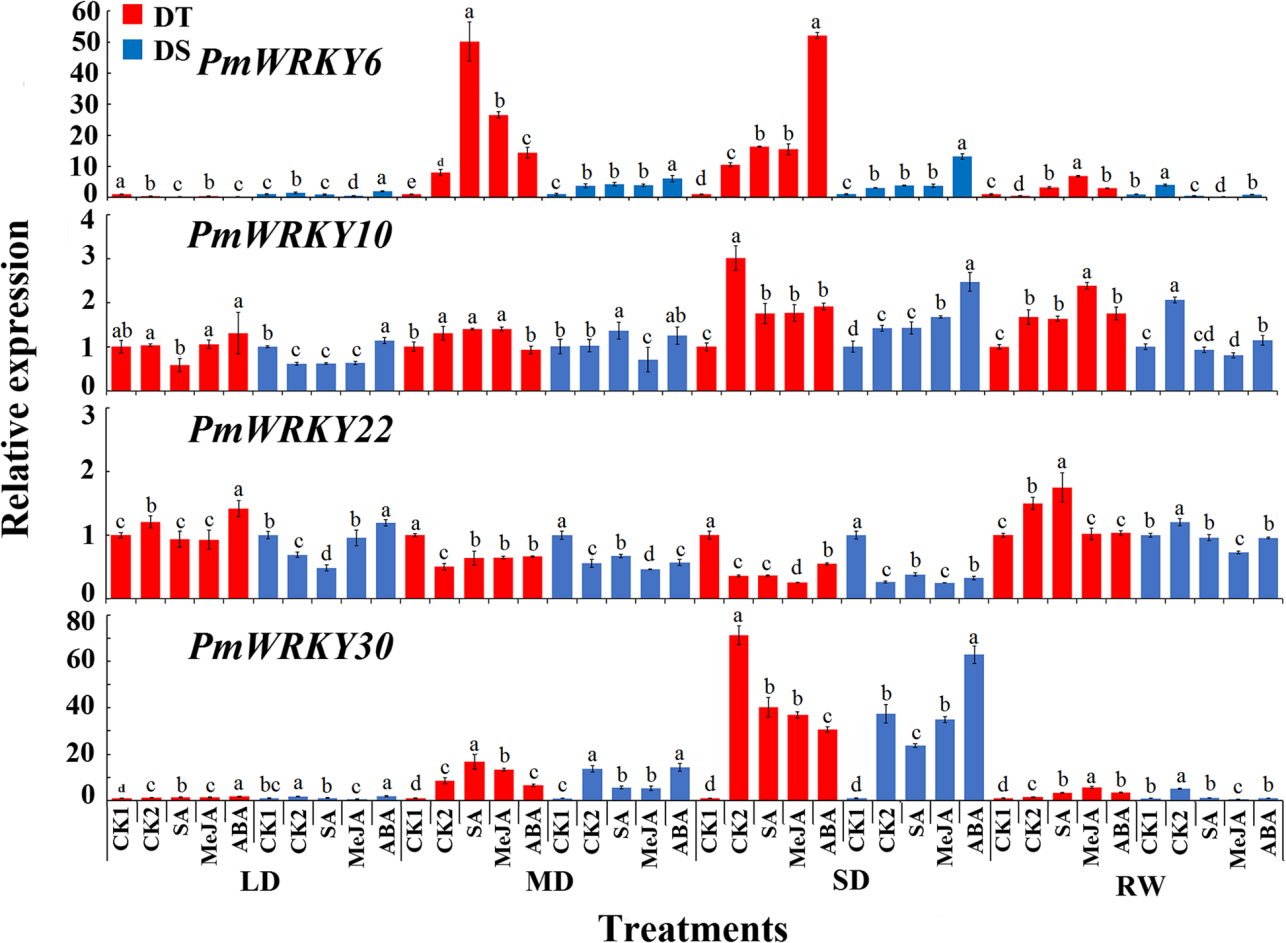
Expression patterns of *PmWRKYs* in stems of the drought-tolerant and drought-sensitive family during drought stress. LD indicates light drought, MD indicates moderate drought, SD indicates severe drought, and RW indicates rewatering. DT indicates drought-tolerant, DS indicates drought-sensitive. Different lowercase letters indicate significant differences at *P* < 0.05

In stems, compared with CK1, expression patterns of 4 *PmWRKYs* were basically same in the two families under drought stress. Expression of *PmWRKY6*, *PmWRKY10* and *PmWRKY30* was up-regulated, while the expression of *PmWRKY22* was down-regulated. Expression of *PmWRKY6* in stems of DT was much higher than that in DS stems. Compared with CK2, there were different expression patterns for these 4 genes in different drought resistant families. Expression of *PmWRKY6* was induced by SA, MeJA and ABA in DT, and expression of 4 genes was significantly induced by ABA in DS. After re-watering, compared with CK1, only expression of *PmWRKY30* was down-regulated in drought-resistant families CK2, while the other treatments showed that expression levels of 4 genes in CK2 were significantly higher than those in CK1 and higher than those in hormone treatments (Fig. 6).

**Fig. 7:**
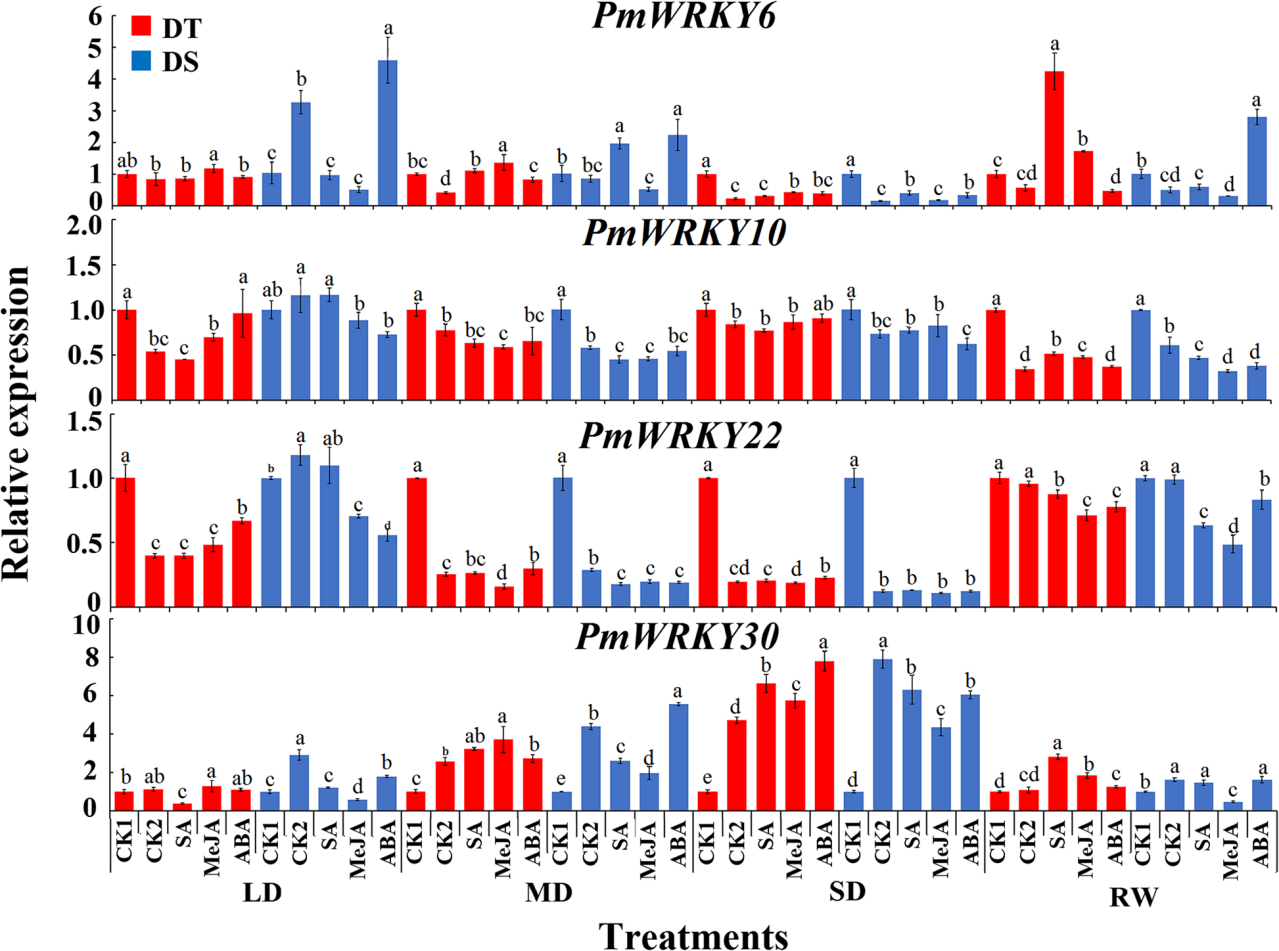
Expression patterns of *PmWRKYs* in roots of the drought-tolerant and drought-sensitive family during drought stress. LD indicates light drought, MD indicates moderate drought, SD indicates severe drought, and RW indicates rewatering. DT indicates drought-tolerant, DS indicates drought-sensitive. Different lowercase letters indicate significant differences at *P* < 0.05

In roots, compared with CK1, expression of *PmWRKY30* was up-regulated in the two families. Expression levels of *PmWRKY6*, *PmWRKY10* and *PmWRKY22* were higher than those of CK1 during light drought stress in DS, while all three genes were down-regulated in DT. Compared with CK2, *PmWRKY6* was up-regulated by MeJA and ABA in DS, and was up-regulated by SA and MeJA in DT. *PmWRKY10* and PmWRKY22 were up-regulated by MeJA in DS. In DT, *PmWRKY10* was up-regulated by MeJA and ABA, and *PmWRKY22* was up-regulated by ABA. *PmWRKY30* was up-regulated by three hormones in DT, and only up-regulated by ABA in DS. After re-watering, *PmWRKY6* and *PmWRKY30* were up-regulated by ABA in drought-sensitive families, while these two genes were induced by SA and MeJA in drought-resistant families (Fig. 7).

These 4 *PmWRKYs* were expressed in roots, stems, and leaves, according to results of their expression in response to hormone and drought stress in each tissue of the two families. Different *PmWRKYs* responded differently to hormone signals. There might be different hormone pathways in response to drought stress in different drought-resistant families. It was speculated that the expression of *PmWRKY6*, *PmWRKY10* and *PmWRKY30* could be involved in response to drought stress.

### Phylogenetic analysis of PmWRKY31 gene information and overexpression in tobacco under drought stress

**Fig. 8:**
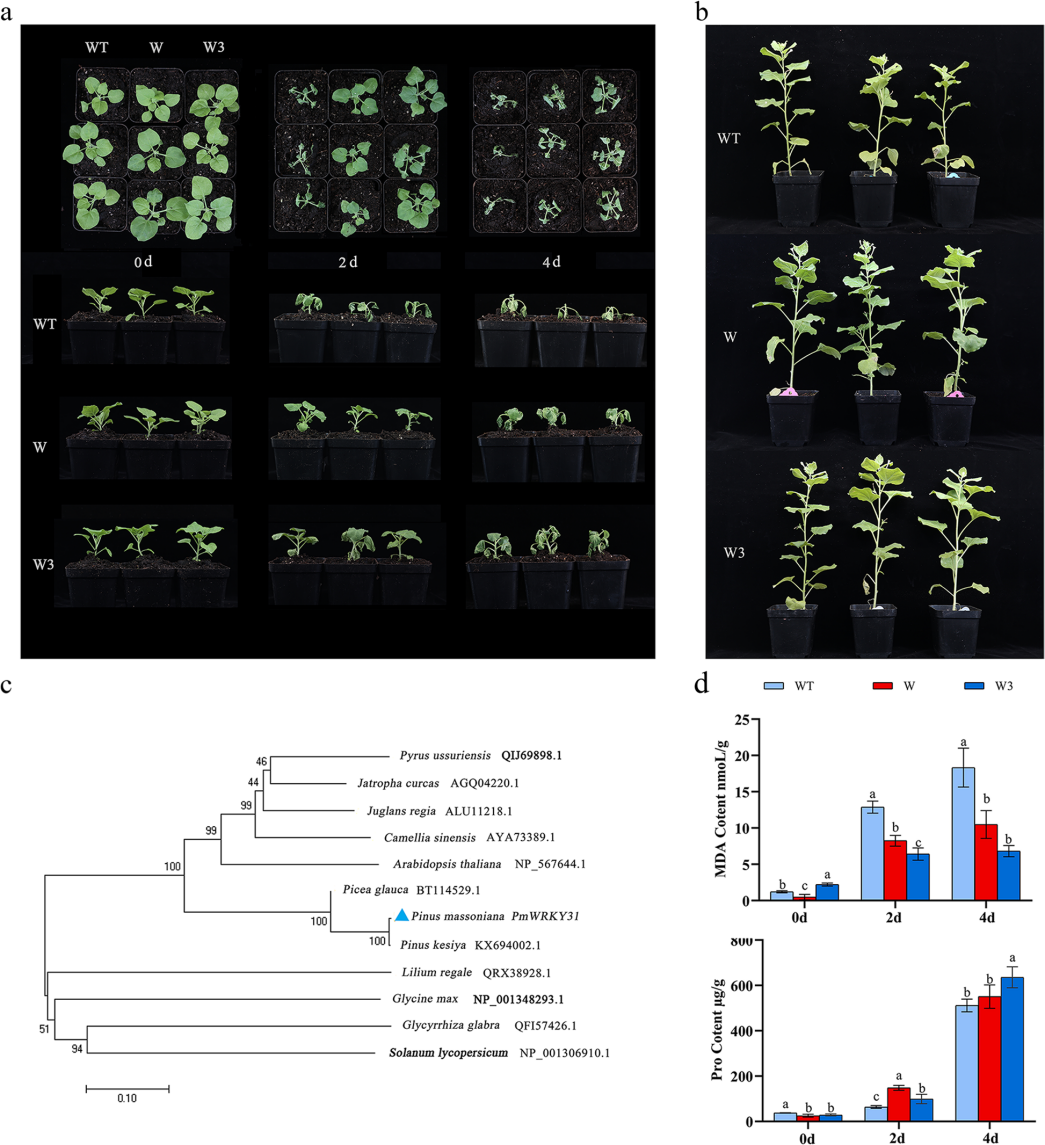
Functional verification of *PmWRKY31*-overexpressing transgenic tobacco. **a** Phenotypic changes in tobacco under drought stress. **b** Phenotypes of WT tobacco and the transgenic W and W3 lines at 60 d of normal culture. **c** Phylogenetic tree analysis of WRKY genes in different species. The blue triangle represents PmWRKY31. **d** Changes in the contents of MDA and Pro under drought stress. 0 d, 2 d, and 4 d represent the drought stress treatment time. WT, W, and W3 represent wild-type tobacco and the W and W3 lines of transgenic tobacco, respectively. Different lowercase letters indicate significant differences at *P* < 0.05

Our research group previously analysed the insect resistance mechanism of *PmWRKY31* (*PmWRKY30* in this paper) [28] and found, through transcriptome data, that *PmWRKY31* was upregulated under drought conditions. Therefore, the drought tolerance function of *PmWRKY31* was verified by overexpressing *PmWRKY31* in tobacco (Fig. 8). After the simulated drought treatment, the phenotypic changes in water loss and wilting were obvious in both transgenic tobacco and WT tobacco (Fig. 8a). Under normal culture conditions, there was no difference in the growth status of transgenic tobacco and wild-type (WT) tobacco (Fig. 8b). On the 2nd day of the stress treatment, both transgenic and WT tobacco plants exhibited significant changes. The leaves of the WT tobacco plants were severely wilted, and the upper leaves of the transgenic tobacco plants of the W and W3 lines grew normally. On the 4th day of stress treatment, WT tobacco plants were severely wilted, with significant water loss in the stems, while the transgenic plants only exhibited wilting leaves. The results indicate that *PmWRKY31* improved the drought tolerance of tobacco plants (Fig. 8a).

The phylogenetic tree showed that *P. massoniana* and *Pinus kesiya* had the closest genetic relationship. The similarity between *PmWRKY31* and the amino acid sequences of other plants was between 41.54% and 99.53%, and the homology between *PmWRKY31* and *Pinus kesiya* was highest, which is consistent with the phylogenetic tree, indicating significant differences in the protein structures of the same gene family between gymnosperms and angiosperms (Fig. 8c).

### Changes in MDA and Pro contents in tobacco plants overexpressing *PmWRKY31* under drought stress

Before treatment (0 d), the MDA content in the W3 line was the highest. During the drought stress process, the MDA content of the WT was significantly increased and was significantly higher than that of the transgenic tobacco (*P* < 0.05). This result indicates that *PmWRKY31* further increased the drought tolerance of tobacco by reducing MDA accumulation (Fig. 8d). The Pro content in the transgenic tobacco was higher than that in WT tobacco during the drought stress process. The results indicated that under drought stress, *PmWRKY31* improved drought tolerance by promoting Pro accumulation (Fig. 8d).

### Analysis of the gene expression patterns in tobacco plants overexpressing *PmWRKY31* under drought stress

**Fig. 9:**
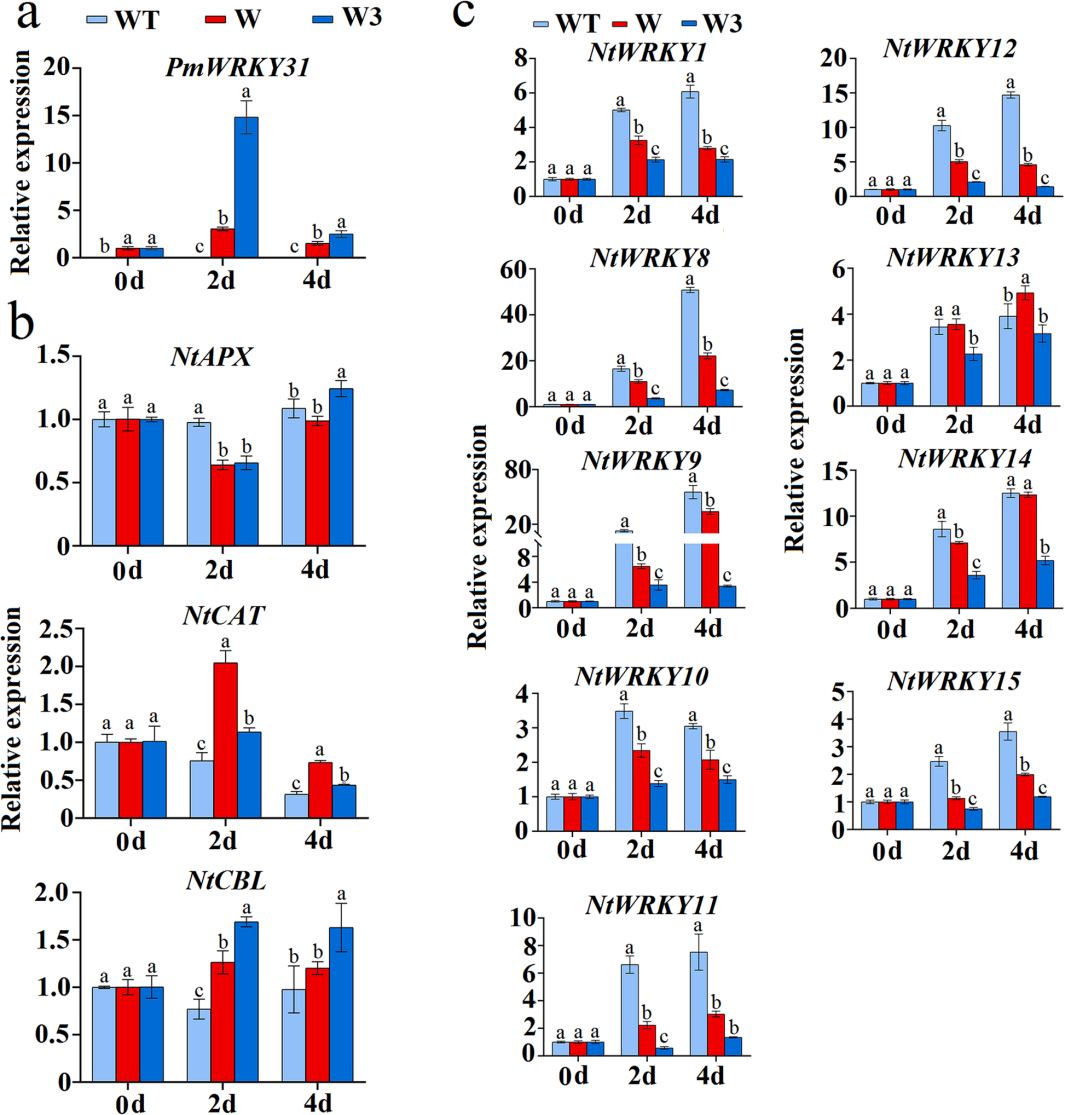
Gene expression analysis of *PmWRKY31*-overexpressing transgenic tobacco and WT tobacco under drought stress. **a** The expression pattern of *PmWRKY31* in tobacco under drought stress. **b** The expression patterns of drought tolerance-related genes, i.e., *NtAPX*, *NtCBL* and *NtCAT*, in tobacco under drought stress. **c** The expression patterns of *NtWRKY*s in tobacco under drought stress. 0 d, 2 d, and 4 d represent the drought stress treatment time. WT, W, and W3 represent wild-type tobacco and the W and W3 lines of transgenic tobacco, respectively. *NtAPX*, *NtCBL*, *NtCAT* and *NtWRKY*s for tobacco were downloaded from PlantTFDB and the NCBI database. Different lowercase letters indicate significant differences at *P* < 0.05

During drought stress, no *PmWRKY31* expression was detected in WT tobacco (Fig. 9a). Under drought stress, *PmWRKY31* expression first increased and then decreased in the W and W3 lines. *PmWRKY31* expression in the W3 line was higher than that in the W line during the stress process (Fig. 9a).

The expression of the *NtAPX*, *NtCBL* and *NtCAT* genes in tobacco was upregulated overall, but the expression patterns were different. The expression of *NtAPX* was upregulated on the 4th day of treatment, the expression of *NtCBL* was upregulated during the entire stress process, and the expression of *NtCAT* decreased after the initial increase but was still higher than that of WT tobacco (Fig. 9b). These results indicated that *PmWRKY31* might enhance the drought tolerance of transgenic tobacco by regulating downstream drought tolerance-related genes.

In addition, under drought stress, the expression of *NtWRKYs* in WT tobacco, except for 2 genes, decreased on the 4th day of treatment, and the expression of other genes was upregulated during the entire stress process and was higher than their expression in W and W3. These results indicate that *PmWRKY31* inhibits the expression of *NtWRKY*s in tobacco (Fig. 9c).

## Discussion

With the development of omics technology, more species of TF families have been identified. A large number of studies have shown that the WRKY TF family is involved in plant growth and development and plant response to abiotic stress [29, 30]. In this study, 43 *PmWRKYs* were identified based on transcriptome data. The number of identified *PmWRKYs* was lower than the 72 *WRKYs* in *A. thaliana* [5], 104 *WRKYs* in *P. Trichocarpa* [7], 61 *WRKYs* in *Taxus chinensis* (also a gymnosperm) [31] but was higher than the reported 25 [23] or 31 *WRKYs* [32] in *P. massoniana*, indicating that this study is more comprehensive. This study revealed that PmWRKYs had 4 types of variations in the conserved heptapeptide structure. The variations included structural variations, which may cause the genes to be unable to specifically bind to downstream genes, thereby losing gene function. For example, in *Crystalline soybean*, GmWRKY6 and GmWRKY21 with variations in the heptapeptide structure cannot bind to the downstream gene W-box [33]. In addition, zinc finger deletion (PmWRKY36) and mutation (PmWRKY39 and PmWRKY43) occurred in PmWRKY proteins, but whether the function of the zinc finger structure (such as PPI and assisting DNA binding) is lost in these 3 genes still needs to be further studied [8]. Conserved motif analysis showed that the conserved motifs of genes in the same subfamily were basically the same, suggesting that genes in the same subfamily might have similar functions [25]. In this study, the PmWRKY gene family was classified into families I, II and III. Family II was categorized into 5 subfamilies. The phylogenetic tree shows that subfamilies IIa and IIb have a close genetic relationship, that subfamilies IId and IIe are clustered on the same branch, and that subfamily IIc is far from the above 4 subfamilies and has evolutionary characteristics similar to those of family II of *A. thaliana* [34]. Furthermore, the subfamilies in *P. massoniana* had closer relationships with gymnosperms, indicating that the evolutionary relationship between gymnosperms was close. In summary, the study results indicate that the WRKY gene family of *P. massoniana* is highly conserved with regard to gene structure and evolutionary level.

In this study, under drought stress and hormone induction, the 4 *PmWRKYs* were constitutively expressed under drought treatment, *PmWRKY6, PmWRKY10* and *PmWRKY30* positively regulated drought stress, and *PmWRKY22* negatively regulated drought stress. It was reported that *MaWRKYs* enhance the cold tolerance of bananas by combining the W-box elements of *MaNCED1* and *MaNCED2* [35]. Whether each *PmWRKY* acts alone or multiple *PmWRKYs* act together in response to drought stress needs to be further investigated. Combined with the expression results for *PmWRKYs* in families with different drought tolerance, it is speculated that *PmWRKY6* and *PmWRKY30* are the key genes that enhance the response of the drought-tolerant family to drought.

Hormones play a vital role in the growth and development and abiotic stress response of plants. For example, SA, ABA, and JA signals can independently interact with each other to improve the drought tolerance of plants [36, 37]. For example, TFs can improve plant stress resistance by participating in SA signalling, *AtWRKY39* overexpression in *Arabidopsis* can improve its heat tolerance, and *AtWRKY39* expression is induced by SA and MeJA to improve plant heat tolerance [38]. *ZmWRKY79* enhances drought tolerance in plants by regulating the synthesis of ABA [39]. The above findings indicate that TFs can play a role by participating in hormone synthesis or by participating in the hormone signal transduction network. In this study, under drought stress, *PmWRKYs* responded to exogenous SA, ABA, and MeJA, and each *PmWRKY* responded to at least 1 hormone, indicating that *PmWRKYs* may improve drought tolerance in *P. massoniana*. *JrWRKY6* and *JrWRKY53* in *Juglans regia* may enhance its resistance to salinity, drought and heat stress through the ABA signalling pathway and W-box binding activity [39]. *CsWRKY2* in *Camellia sinensis* is involved in the ABA downstream signalling pathway and improvements in drought tolerance [40]. In this study, *PmWRKY6* and *PmWRKY30* expression was upregulated by treatment with 3 hormones. It is speculated that *PmWRKY6* and *PmWRKY30* may be involved in a hormone signalling pathway and improve the drought tolerance of *P. massoniana*. However, the specific mechanisms need to studied further.

Interestingly, although the expression of *PmWRKY22* was induced by hormones, its expression was lower than that of CK1. Studies have shown that *AtWRKY18*, *AtWRKY40*, and *AtWRKY60* are negative regulators of ABA signalling and that these genes, as the upstream genes of the ABA signalling pathway, participate in the ABA signalling regulatory network by inhibiting ABA signalling pathway-related genes such as *MYB2* and *ABI5* [41, 42]. Therefore, it is speculated that *PmWRKY22* may negatively regulate hormonal signals in response to drought. The expression of *PmWRKYs* induced by hormone signals was different in the 2 families. This result was similar to the expression pattern of *CsPRX*s in 2 varieties of *C. sinensis* [43], results that may be caused by the different genetic backgrounds of the 2 families with different drought tolerance. In addition, it is reasonable to believe that the high expression of *PmWRKYs* increases the drought tolerance of the drought-tolerant family of *P. massoniana*.

Currently, in plants with no established genetic transformation system, the effects of genes on plant phenotypes, physiology, and related genes are analysed through functional validation in model plants to explore the functions of genes in improving plant resistance [30, 44]. In this study, *PmWRKY31*-overexpressing transgenic tobacco was more drought tolerant than WT tobacco; its MDA content was significantly lower than that of WT tobacco, and its Pro accumulation was higher than that of WT tobacco. The results indicate that transgenic tobacco could better alleviate membrane damage and maintain osmotic regulation and thereby has better drought tolerance [17, 20].

TFs mainly regulate downstream genes, activate or inhibit the expression of downstream genes, and interact with drought-related genes to resist drought stress [17, 45]. *PbrWRKY53* improves drought tolerance of tobacco by increasing the expression of drought tolerance-related genes such as *NtSOD and NtCAT* [46]. In this study, the expression of *NtCBL*, *NtCAT*, and *NtAPX* in transgenic tobacco was upregulated, indicating that *PmWRKY31* might increase the drought tolerance of tobacco by increasing the expression of drought stress-related genes. Interestingly, *PmWRKY31* inhibited the expression of *NtWRKY*s in *PmWRKY31*-overexpressing transgenic tobacco to improve drought tolerance, but the specific regulatory mechanism needs to be further studied.

## Conclusions

In this study, we successfully identified 43 PmWRKYs and analysed the physicochemical properties, phylogenetic evolution, and conserved motifs of PmWRKY genes family members. We found that the genes of this family have specific domains with highly conserved evolutionary levels. According to transcriptome data and gene expression analysis, *PmWRKYs* respond to drought stress and phytohormone treatments at different degrees and stress stage. It is also found that *PmWRKYs* in response to drought stress and phytohormones might have the same or different regulatory mechanisms. Furthermore, the transformation tobaccos of *PmWRKY31* show that by regulating the expression of *NtCBL*, *NtCAT* and *NtAPX*, MDA content decreases, Pro content increases, and drought resistance is improved by alleviating membrane system damage and maintaining osmoregulation. These will contribute to further study on molecular mechanism of *PmWRKYs* in response to drought in *P. massoniana*.

## Materials and methods

### Plant material and drought stress treatments

The drought-tolerant family 19–220 (DT) and the drought-sensitive family 19–214 (DS) of *P. massoniana* were provided by the Timber Forest Research Institute, Guangxi Forestry Research Institute (GFRI) Nanning, China. Two germplasm were selected from 132 excellent families by treatments of simulating natural drought stress, and then comprehensive evaluation of wilting degree, survival rate, seedling height, ground diameter and other indicators. We had got the permission to collect and use the two germplasm lines which were stored in GFRI. The experimental research and field studies on plants comply with relevant institutional, national, and international guidelines and legislation. Half-year-old seedlings of the 2 families with a good and basically consistent growth status were selected and placed in the drought shed of the nursery. Based on the results of previous experiments (Supplementary Table 2, Supplementary Fig. 2), 50 mg/L SA, 0.5 mmol/L MeJA, and 25 mg/L ABA were used for spraying treatments. Five groups were set up, i.e., CK1, normal watering; CK2, drought stress; 50 mg/L SA + drought stress; 0.5 mmol/L MeJA + drought stress; 25 mg/L ABA + drought stress, with 3 replicates for each treatment and 10 plants for each replicate. After 3 days of continuous watering, the above concentrations of SA, MeJA, ABA and distilled water (CK1 and CK2) were sprayed on the plants, with 10 mL sprayed on the aboveground part (stems and needles) and 10 mL sprayed on the underground part (roots). After spraying at 8 am every day for 4 consecutive days, all experimental seedlings were supplemented with 20 mL of water, which was recorded as the 0th day of drought stress, and the seedlings were subjected to continuous natural drought stress. CK1 was the normal watering control group, and the seedlings in this group were watered once every 2 days. The needles (2 cm from the top of the plant), stems (3.5 cm from the tip of the stem), and roots (3 cm from the tips of the main root and lateral roots) of P. massoniana were collected at light drought stress (LD, 55–70%, soil moisture content, MC %), moderate drought stress (MD, 45–55%), severe drought stress (SD, 30–45%) [47], and 48 h after re-watering (RW) and stored in a -80 °C freezer. The classification of drought grade and the determination of soil moisture content during sampling were determined according to the treatment phenotype of plants, as shown in Supplementary Fig. 2.

WT tobacco (*Nicotiana benthamiana*) and the F4 generation lines W and W3 of *PmWRKY31*-overexpressing transgenic tobacco were used as experimental materials. Seeds of WT tobacco were provided by Gold Tech Biotechnology Co., LTD (Nanning China). WT tobacco leaves were used as material to induce sterile tobacco seedlings, and pBI121_*PmWRKY31* was constructed for Agrobacterium transformation. Hygromycin was used for pre-screening. DNA of seedlings from transgenic tobaccos was extracted by CTAB method from leaves. According the sequence of *PmWRKY31*, PCR primers were designed for specific detection (PmWRKY31F:CCCTCTAGAATGGAAGCAGTGGGGTTGAG, PmWRKY31R: TTTCCCGG GTCACTGGAGCAATTTAGCC). Transgenic Tobaccos with *PmWRKY31* was cultured to F4 generation for further experiment [28]. They were cultured for 6 weeks in an artificial climate chamber (25 °C during the daytime/28 °C at night; relative humidity (RH) = 60%; light/dark cycle = 16 h/8 h). Subsequently, polyethylene glycol (PEG) was used to simulate drought stress in both transgenic tobacco and WT tobacco. The specific method was as follows: the roots of each plant were watered with 15 mL of 20% PEG6000, and 1/3 of the 20% PEG6000 was poured into the bottom of the saucer tray to maintain the osmotic state [48]. The phenotypic differences in tobacco were observed. The leaves of transgenic tobacco and WT tobacco were sampled at the same position on the 0th, 2nd, and 4th days of drought stress to assess physiological indicators and the expression of drought tolerance-related genes.

### Identification and physicochemical properties analysis of WRKY family members of *P. massoniana*

*PmWRKY* data were obtained from the third-generation full-length transcriptome and insect-resistant transcriptome of *P. massoniana* sequenced by our research group [49], a transcriptome for lateral branch differentiation [50], transcriptome of Aluminum stress [51], and a drought-tolerant, low-temperature stress, transcriptome (unpublished). Illumina HiSeq 4000 high-throughput sequencer was used for sequencing. The sequencing read length was 150 bp (Sequencing was performed by Novogene Bioinformatics Technology CO., LTD.). Clean Data of all samples was assembled by Trinity software [52]. Unigene sequences were compared with NR, SwisS-ProT, GO, COG, KOG and KEGG databases to obtain the annotation information. Bowtie method [53] and RSEM were used to estimate the expression levels of genes. Reference genomes were Norway spruce (*Picea abies* (L.) Karst.), loblolly pine (*Pinus taeda* L.) and white spruce (*Picea glauca*) as the main species. TransDecoder software was used to predict the coding DNA sequence (CDS). TBtools [54] and the ORF finder (https://www.ncbi.nlm.nih.gov/orffinder) of the National Center for Biotechnology Information (NCBI) were used to predict the longest open reading frame (ORF). The 3 software programs were used together to obtain the nucleic acid sequences and amino acid sequences. Functional annotation was performed using the NR, SwissProt, and Pfam databases. Subsequently, the union of the 3 sets of annotation results was used to extract the nucleic acid and protein sequences using the Perl language. A hidden Markov model of the WRKY gene domain (PF03106) was downloaded from the Pfam database (https://pfam.xfam.org/) [55]. WRKY genes were identified using HMMER software. The amino acid sequences of Arabidopsis WRKY genes downloaded from PlantTFDB (http://planttfdb.gao-lab.org/index_ext.php) [56] were compared with the amino acid sequences of *P. massoniana* WRKY genes to identify protein sequences. CD-HIT (http://weizhong-lab.ucsd.edu/webMGA/server/cd-hit/) was used for clustering analysis, and redundant sequences were removed. NCBI CD Search (https://www.ncbi.nlm.nih.gov/Structure/cdd/wrpsb.cgi) [57], SMART (http://smart.embl.de/) [58] and Pfam were used to detect the WRKY domains of the candidate proteins. Last, *PmWRKYs* of *P. massoniana* were identified by removing the sequences that contained incomplete domains or did not contain domains.

ExPASy (https://web.expasy.org/) [59] was used to predict the physicochemical properties, such as the length of the encoded protein, molecular mass, and pI, of PmWRKYs. Plant-mPLoc (http://www.csbio.sjtu.edu.cn/bioinf/plant-multi/) [60] and WoLF PSORT (https://psort.hgc.jp/) [61] were used for subcellular localization. TMHMM Server v.2.0 (http://www.cbs.dtu.dk/services/TMHMM/) was used to analyse the transmembrane structures of the proteins.

### Phylogenetic analysis, conserved motif analysis and multiple sequence alignment of PmWRKYs

The WRKY gene protein sequences of *Arabidopsis* (72), *Pinus taeda* (20), and *Picea glauca* (16) were downloaded from PlantTFDB. MEGA 7 software [62] was used to perform multiple sequence alignment of the WRKY protein sequences of *P. massoniana*, *A. thaliana*, *P. taeda*, and *P. glauca*. The neighbour-joining (NJ) method was used to reconstruct phylogenetic trees using the P-distance model through 1000 bootstrap iterations. Subsequently, iTOL [63] was used to improve the phylogenetic trees. MEME (https://meme-suite.org/meme/) [64] was used to analyse the conserved motifs of the WRKY gene of *P. massoniana* with the following parameter settings: number of predicted motifs = 10, and length of the motifs = 6–60 AA. ClustalW software was used to perform multiple sequence alignments of WRKY proteins in each subfamily, and GeneDoc software was used to generate multiple sequence alignment diagrams.

### Analysis of protein interaction among PmWRKYs

The STRING website (https://string-db.org/cgi) [65] and Cytoscape software [66] were used to predict the potential PPI network and biological functions of PmWRKY proteins in *P. massoniana* based on the WRKY protein analysis of *A. thaliana*.

### RNA-Seq data analysis of *PmWRKYs*

An expression heatmap of *PmWRKYs* under drought stress was generated based on previous drought transcriptome data. Transcriptome data were sequenced by double-ended sequencing (PE150) using an Illumina HiSeq 4000 sequencer, and the resulting clean reads were assembled using Trinity software to obtain spliced transcripts (Unigene) for subsequent analysis. A treatment group (D) and control group (C) were set up. The treatment group was under continuous natural drought stress, and the control group was normally watered. The roots of the seedlings of the treatment group and the control group were collected on the 7th day (D1), 7 h after rewatering on the 7th day (D2), and 8th day (D3) of drought stress treatmen. For detailed processing and sampling, see Supplementary Fig. 3. Using the obtained transcriptome data, TBtools was used to plot the expression heatmap for the *PmWRKY* gene family under drought stress.

### RNA extraction, cDNA synthesis, and RT–qPCR

RNA was extracted using a polyphenol polysaccharide plant RNA extraction kit from Tiangen (Beijing, China). M-MLV reverse transcriptase (Takara, Shanghai, China) was used for reverse transcription following the specific steps in the instruction manual, reverse transcription primer was Oligo(dT)_18_:5'-GGCCACGCGTCGACTAG TAC(T)_18_–3'. The concentration of all cDNA samples was adjusted to 50 ng/μL. Primer Premier 5.0 was used to design fluorescent quantitative PCR primers (Supplementary Table 3) with *PmCYP* as the internal reference gene [67], Nine *N. benthamiana* gene sequences were downloaded from NCBI and Adachi et al. [68]. A TB Green ® Premix Ex Taq™ II (Tli RNaseH Plus) fluorescence quantification kit (Takara, Shanghai, China) was used to configure the reaction system. PCR analysis was performed using a quantitative PCR system (CFX96, Bio–Rad, California, USA). The measurement was biological replicated 3 times for each sample, and the relative expression of each gene was calculated using the 2^−ΔΔCt^ method [69].

### Data analysis

One-way analysis of variance (ANOVA) in SPSS software was used to analyse the significance of the data. TBtools was used to generate the expression heatmap, and GraphPad Prism 8 was used to plot histograms.

## Supplementary Information

**Additional file 1: Supplementary Fig 1.** Sequence alignment of WRKY conserved domain proteins in *P.* *massoniana*. The horizontal line represents the WRKY conserved heptapeptide domain. The blue arrow indicates the zinc finger structure.

**Additional file 2: Supplementary Fig. 2.** The phenotypic changes of *P. massoniana* seedlings treated with different concentrations of exogenous hormones.

**Additional file 3: Supplementary Fig. 3.** The schematic diagram of drought stress treatment and sampling process of *P. massoniana*.

**Additional file 4: Supplementary Table 1.** Physical and chemical analysis of WRKY in *Pinus massoniana*.

**Additional file 5: Supplementary Table 2.** Effects of exogenous hormones at different concentrations on biomass and root-shoot ratio of *Pinus massoniana* (19–214 and 19–220) under drought stress.

**Additional file 6: Supplementary Table 3.** qRT-PCR Primer sequences of the genes.

## Data Availability

The data archiving statement of high-throughput sequencing and raw data are available in the NCBI GEO database accession, [GSE72294] (https://www.ncbi.nlm.nih.gov/gds/?term=GSE72294).

## References

[1] Shinozaki K, Kazuko Y, Motoaki S (2003). Regulatory network of gene expression in the drought and cold stress responses. Curr Opin Plant Biol.

[2] Singh D, Debnath P, Roohi, Sane AP, Sane VA (2020). Expression of the tomato WRKY gene, *SlWRKY23*, alters root sensitivity to ethylene, auxin and JA and affects aerial architecture in transgenic *Arabidopsis*. Physio Mol Biol Plants..

[3] Yu Y, Qi Y, Xu J, Dai X, Chen J, Dong C (2021). *Arabidopsis WRKY71* regulates ethylene-mediated leaf senescence by directly activating *EIN2*, *ORE1* and *ACS2* genes. Plant J.

[4] Ishiguro S, Nakamura K (1994). Characterization of a cDNA encoding a novel DNA-binding protein, SPF1, that recognizes SP8 sequences in the 5' upstream regions of genes coding for sporamin and β-amylase from sweet potato. Mol Gen Genet.

[5] Lker BU, Imre ES (2004). WRKY transcription factors: from DNA binding towards biological function. Curr Opin Plant Biol.

[6] Ross CA, Liu Y, Shen QJ (2007). The WRKY gene family in rice (*Oryza sativa*). J Integr Plant Biol.

[7] He H, Qing D, Shao Y, Jiang H, Zhu S, Cheng B (2012). Genome-wide survey and characterization of the WRKY gene family in *Populus trichocarpa*. Plant Cell Rep.

[8] Rushton PJ, Imre ES, Patricia R, Qingxi JS (2010). WRKY transcription factors. Trends Plant Sci.

[9] Maeo K, Hayashi S, Kojima-Suzuki H, Morikami A, Nakamura K (2001). Role of conserved residues of the WRKY domain in the DNA-binding of tobacco WRKY family proteins. Biosci Biotechnol Biochem.

[10] Phukan UJ, Gajendra SJ, Rakesh KS (2016). WRKY transcription factors: molecular regulation and stress responses in plants. Front Plant Sci.

[11] Ciolkowski I, Wanke D, Birkenbihl RP, Somssich IE (2008). Studies on DNA-binding selectivity of WRKY transcription factors lend structural clues into WRKY-domain function. Plant Mol Biol.

[12] Mangelsen E, Kilian J, Berendzen KW, Kolukisaoglu ÜH, Harter K, Jansson C (2008). Phylogenetic and comparative gene expression analysis of barley (*Hordeum vulgare*) WRKY transcription factor family reveals putatively retained functions between monocots and dicots. BMC Genomics.

[13] Sun C, Palmqvist S, Olsson H, Borén M, Ahlandsberg S, Jansson C (2003). A novel WRKY transcription factor, SUSIBA2, participates in sugar signaling in barley by binding to the sugar-responsive elements of the *iso1* promoter. Plant Cell.

[14] Cai M, Qiu D, Yuan T, Ding X, Li H, Liu D (2008). Identification of novel pathogen-responsive *cis*-elements and their binding proteins in the promoter of *OsWRKY13*, a gene regulating rice disease resistance. Plant Cell Environ.

[15] Zhang Q, Zhu J, Ni Y, Cai Y, Zhang Z (2012). Expression profiling of *HbWRKY1*, an ethephon-induced *WRKY* gene in latex from *Hevea brasiliensis* in responding to wounding and drought. Trees.

[16] Wang Z, Feng R, Zhang X, Su Z, Wei J, Liu J (2019). Characterization of the *Hippophae rhamnoides WRKY* gene family and functional analysis of the role of the *HrWRKY21* gene in resistance to abiotic stresses. Genome.

[17] Chu X, Wang C, Chen X, Lu W, Li H, Wang X (2017). The cotton WRKY gene *GhWRKY41* positively regulates salt and drought stress tolerance in transgenic *Nicotiana benthamiana*. PLoS ONE.

[18] Wang L, Wang C, Qin L, Hu P, Wang Y (2016). *ThERF1* from *Tamarix hispida* confers decreased tolerance to oxidative and drought stresses and is regulated by a WRKY protein. J For Res.

[19] Wang D, Chen Q, Chen W, Liu X, Yan X, Qigao G (2021). A WRKY transcription factor, *EjWRKY17*, from *Eriobotrya japonica* enhances drought tolerance in transgenic *Arabidopsis*. Int J Mol Sci.

[20] Li Z, Liang F, Zhang T, Fu N, Pei X, Long Y (2021). Enhanced tolerance to drought stress resulting from *Caragana korshinskii CkWRKY33* in transgenic *Arabidopsis thaliana*. BMC Genom Data.

[21] Ye H, Linyi Q, Haoyu G, Liping G, Fei R, Jianfang B (2021). Genome-wide identification of wheat *WRKY* gene family reveals that *TaWRKY75-A* is referred to drought and salt resistances. Front Plant Sci.

[22] Li M, Wang H, Zhao X, Lu Z, Sun X, Ding G (2021). Role of Suillus Placidus in improving the drought tolerance of Masson Pine (Pinus massoniana Lamb.) seedlings. Forests..

[23] Yao S, Wu F, Hao Q, Ji K (2020). Transcriptome-wide identification of WRKY transcription factors and their expression profiles under different types of biological and abiotic stress in *Pinus massoniana* Lamb. Genes.

[24] Shao C, Duan H, Ding G, Luo X, Fu Y, Lou Q (2022). Physiological and biochemical dynamics of Pinus massoniana Lamb. seedlings under extreme drought stress and during recovery. Forests..

[25] Eulgem T, Paul JR, Silke R, Imre ES (2000). The WRKY superfamily of plant transcription factors. Trends Plant Sci.

[26] Wu K, Guo Z, Wang H, Li J (2005). The WRKY family of transcription factors in rice and *Arabidopsis* and their origins. DNA Res.

[27] Jiang Y, Deyholos MK (2009). Functional characterization of *Arabidopsis* NaCl-inducible *WRKY25* and *WRKY33* transcription factors in abiotic stresses. Plant Mol Biol.

[28] Chen H, Hu Y, Liang X, Xie J, Xu H, Luo Q, et al. Roles of hormones, calcium and *PmWRKY31* in the defense of *Pinus massoniana* Lamb. against *Dendrolimus punctatus* Walker. Forestry Research. 2021;1:1–14. 10.48130/FR-2021-0021. 10.48130/FR-2021-0021 PMC11524255 39524514

[29] Gan Z, Yuan X, Shan N, Wan C, Chen C, Xu Y (2021). *AcWRKY40* mediates ethylene biosynthesis during postharvest ripening in kiwifruit. Plant Sci.

[30] Ullah A, Sun H, Hakim, Yang X, Zhang X (2018). A novel cotton WRKY gene, *GhWRKY6-like*, improves salt tolerance by activating the ABA signaling pathway and scavenging of reactive oxygen species. Physiol Plant.

[31] Zhang M, Chen Y, Nie L, Jin X, Liao W, Zhao S (2018). Transcriptome-wide identification and screening of WRKY factors involved in the regulation of taxol biosynthesis in *Taxus chinensis*. Sci Rep.

[32] Fan F, Wang Q, Li H, Ding G, Wen X (2021). Transcriptome-wide identification and expression profiles of Masson Pine WRKY transcription factors in response to low phosphorus stress. Plant Mol Biol Rep.

[33] Zhou Q, Tian A, Zou H, Xie Z, Lei G, Huang J (2008). Soybean WRKY-type transcription factor genes, *GmWRKY13*, *GmWRKY21*, and *GmWRKY54*, confer differential tolerance to abiotic stresses in transgenic *Arabidopsis* plants. Plant Biotechnol J.

[34] Zhang Y, Wang L (2005). The WRKY transcription factor superfamily: its origin in eukaryotes and expansion in plants. BMC Evol Biol.

[35] Luo D, Ba L, Shan W, Kuang J, Lu W, Chen J (2017). Involvement of WRKY transcription factors in abscisic-acid-induced cold tolerance of banana fruit. J Agr Food Chem.

[36] Yang G, Zhang W, Sun Y, Zhang T, Hu D, Zhai M (2017). Two novel WRKY genes from *Juglans regia*, *JrWRKY6* and *JrWRKY53*, are involved in abscisic acid-dependent stress responses. Biol Plant.

[37] Puranik S, Kumar K, Srivastava PS, Prasad M (2011). Electrophoretic mobility shift assay reveals a novel recognition sequence for *Setaria italica* NAC protein. Plant Signal Behav.

[38] Li S, Zhou X, Chen L, Huang W, Yu D (2010). Functional characterization of *Arabidopsis thaliana WRKY39* in heat stress. Mol Cells.

[39] Gulzar F, Fu J, Zhu C, Yan J, Lin X, Meraj T (2021). Maize WRKY transcription factor *ZmWRKY79* positively regulates drought tolerance through elevating ABA biosynthesis. Int J Mol Sci.

[40] Wang Y, Shu Z, Wang W, Jiang X, Li D, Pan J (2016). *CsWRKY2*, a novel WRKY gene from *Camellia sinensis*, is involved in cold and drought stress responses. Biol Plantarum.

[41] Yan L, Liu Z, Xu Y, Lu K, Wang X, Zhang D (2013). Auto- and cross-repression of three *Arabidopsis* WRKY transcription factors *WRKY18*, *WRKY40*, and *WRKY60* negatively involved in ABA signaling. J Plant Growth Regul.

[42] Shang Y, Yan L, Liu Z, Cao Z, Mei C, Xin Q (2010). The Mg-chelatase H subunit of *Arabidopsis* antagonizes a group of WRKY transcription repressors to relieve ABA-responsive genes of inhibition. Plant Cell.

[43] Li H, Wang H, Chen Y, Ma Q, Zhao Z, Li X (2020). Isolation and expression profiles of class III *PRX* gene family under drought stress in *Camellia sinensis*. Biol Plant Arum.

[44] Zhu H, Jiang Y, Guo Y, Huang J, Zhou M, Tang Y (2021). A novel salt inducible WRKY transcription factor gene, *AhWRKY75*, confers salt tolerance in transgenic peanut. Plant Physiol Bioch.

[45] Dong Q, Zheng W, Duan D, Huang D, Wang Q, Liu C (2020). *MdWRKY30*, a group IIa WRKY gene from apple, confers tolerance to salinity and osmotic stresses in transgenic apple callus and *Arabidopsis* seedlings. Plant Sci.

[46] Liu Y, Yang T, Lin Z, Gu B, Xing C, Zhao L (2019). A WRKY transcription factor *PbrWRKY53* from *Pyrus betulaefolia* is involved in drought tolerance and AsA accumulation. Plant Biotechnol J.

[47] Du M, Ding G, Cai Q (2018). The transcriptomic responses of *Pinus massoniana* to drought dtress. Forests.

[48] Chen H, Zhou J, Li Y, Yang H, Lu X, Ma W (2007). I dentificati on of drought resistance of flue cured Tobacco with PEG. J Henan Agri Sci.

[49] Yang Z, Chen H, Jia J, Luo Q, Tang S, Li K (2016). De novo assembly and discovery of metabolic pathways and genes that are involved in defense against pests in Songyun *Pinus massoniana* Lamb. Bangladesh J Bot.

[50] Chen H, Tan J, Liang X, Tang S, Jia J, Yang Z (2021). Molecular mechanism of lateral bud differentiation of *Pinus massoniana* based on high-throughput sequencing. Sci Rep.

[51] Wang T, Hu Y, Chen H, Tan J, Xu H, Li P, Wu D (2022). Transcriptome analysis of response to aluminum stress in *Pinus massoniana*. Forests.

[52] Grabherr MG, Haas BJ, Yassour M, Levin JZ, Thompson DA, Amit IA, et al. Full-length transcriptome assembly from RNA-Seq data without a reference genome. Nat. Biotechnol. 2011;29(7):644–52. 10.1038/nbt.1883 PMC3571712 21572440

[53] Langmead B, Salzberg SL. Fast gapped-read alignment with Bowtie 2. Nat. Methods. 2012;9(4):357–9. 10.1038/nmeth.1923 PMC3322381 22388286

[54] Chen C, Chen H, Zhang Y, Thomas HR, Frank MH, He Y (2020). TBtools: an integrative toolkit developed for interactive analyses of big biological data. Mol Plant.

[55] Mistry J, Chuguransky S, Williams L, Qureshi M, Salazar GA, Sonnhammer ELL (2021). Pfam: the protein families database in 2021. Nucleic Acids Res.

[56] Tian F, Yang D, Meng Y, Jin J, Gao G (2019). PlantRegMap: charting functional regulatory maps in plants. Nucleic Acids Res.

[57] Lu S, Wang J, Chitsaz F, Derbyshire MK, Geer RC, Gonzales NR (2020). CDD/SPARCLE: the conserved domain database in 2020. Nucleic Acids Res.

[58] Letunic I, Khedkar S, Bork P (2021). SMART: recent updates, new developments and status in 2020. Nucleic Acids Res.

[59] Duvaud S, Gabella C, Lisacek F, Stockinger H, Ioannidis V, Durinx C (2021). Expasy, the swiss bioinformatics resource portal, as designed by its users. Nucleic Acids Res.

[60] Chou KC, Shen HB (2010). Plant-mPLoc: a top-down strategy to augment the power for predicting plant protein subcellular localization. PLoS ONE.

[61] Horton P, Park KJ, Obayashi T, Fujita N, Harada H, Adams-Collier CJ (2007). WoLF PSORT: protein localization predictor. Nucleic Acids Res..

[62] Kumar S, Stecher G, Tamura K (2016). MEGA7: molecular evolutionary genetics analysis version 7.0 for bigger datasets. Mol Biol Evol.

[63] Letunic I, Bork P (2021). Interactive tree of life (iTOL) V5: an online tool for phylogenetic tree display and annotation. Nucleic Acids Res.

[64] Bailey TL, Johnson J, Grant CE, Noble WS (2015). The MEME suite. Nucleic Acids Res.

[65] Szklarczyk D, Gable AL, Nastou KC, Lyon D, Kirsch R, Pyysalo S (2021). The STRING database in 2021: customizable protein–protein networks, and functional characterization of user-uploaded gene/measurement sets. Nucleic Acids Res.

[66] Shannon P, Markiel A, Ozier O, Baliga NS, Wang JT, Ramage D (2003). Cytoscape: a software environment for integrated models of biomolecular interaction networks. Genome Res.

[67] Chen H, Yang Z, Hu Y, Tan J, Jia J, Xu H (2016). Reference genes selection for quantitative gene expression studies in *Pinus Massoniana* L. Trees.

[68] Adachi H, Nakano T, Miyagaw N, Ishiham N, Yoshioka M, Katou Y (2015). WRKY transcription factors phosphorylated by MAPK regulate a plant immune NADPH oxidase in *Nicotiana benthamiana*. Plant Cell.

[69] Livak KJ, Schmittgen TD (2001). Analysis of relative gene expression data using real-time quantitative PCR and the 2−ΔΔCT method. Methods.

