# Supplementary material for: Identification of WRKY transcription factor family genes in Pinus massoniana Lamb. and their expression patterns and functions in response to drought stress
Source: BMC Plant Biol. 2022 Sep 1;22:424. doi: 10.1186/s12870-022-03802-7 (PMC9434871; doi:10.1186/s12870-022-03802-7)
Supplement: Supplementary file 1 — Additional file 1: Supplementary Fig 1. Sequence alignment of WRKY conserved domain proteins in P. massoniana. The horizontal line represents the WRKY conserved heptapeptide domain. The blue arrow indicates the zinc finger structure. [file 12870_2022_3802_MOESM1_ESM.docx]

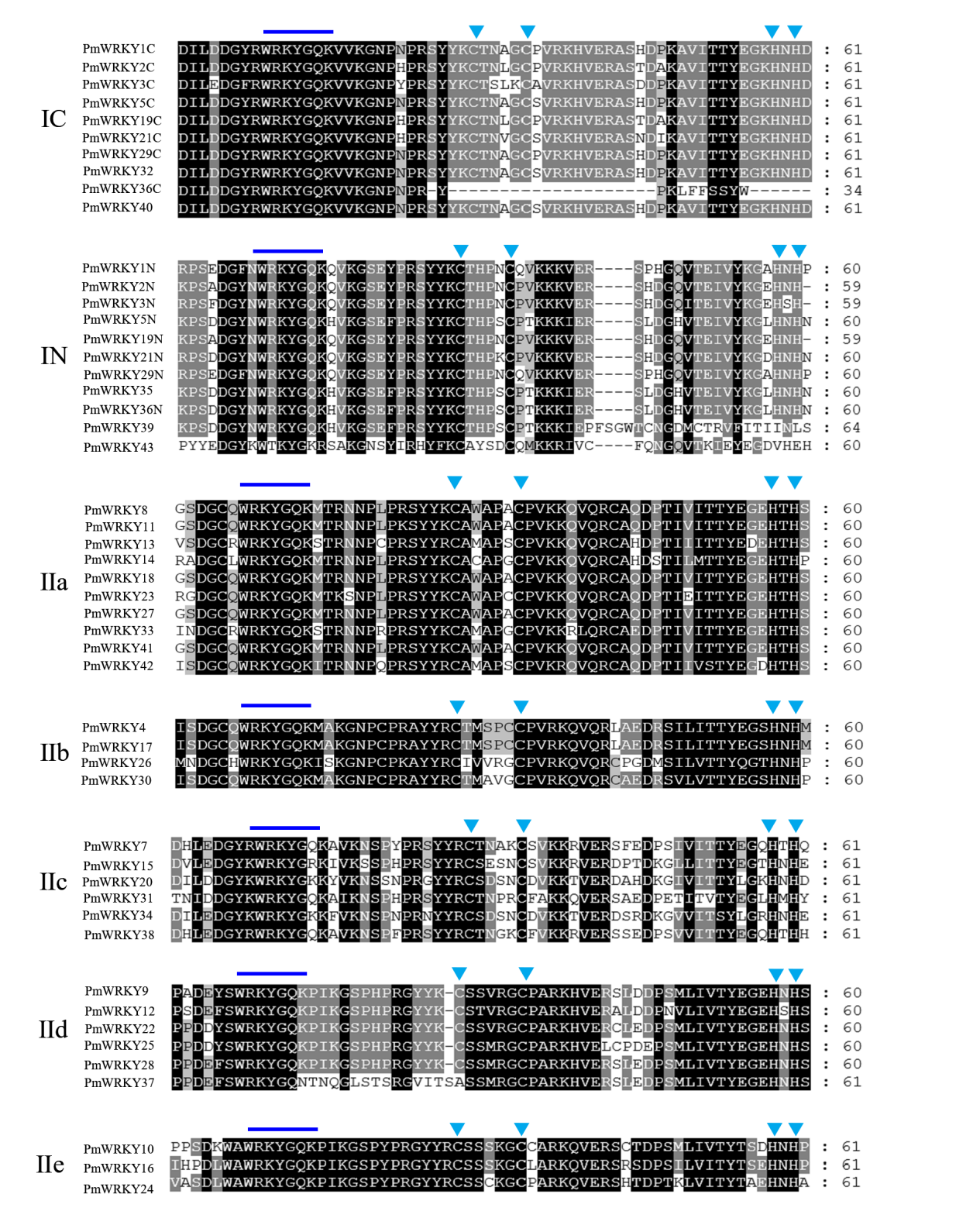


Supplementary Fig 1. Sequence alignment of WRKY conserved domain proteins in *P. massoniana*. The horizontal line represents the WRKY conserved heptapeptide domain. The blue arrow indicates the zinc finger structure.
