# Supplementary material for: Identification of WRKY transcription factor family genes in Pinus massoniana Lamb. and their expression patterns and functions in response to drought stress
Source: BMC Plant Biol. 2022 Sep 1;22:424. doi: 10.1186/s12870-022-03802-7 (PMC9434871; doi:10.1186/s12870-022-03802-7)
Supplement: Supplementary file 2 — Additional file 2: Supplementary Fig. 2. The phenotypic changes of P. massoniana seedlings treated with different concentrations of exogenous hormones. [file 12870_2022_3802_MOESM2_ESM.docx]

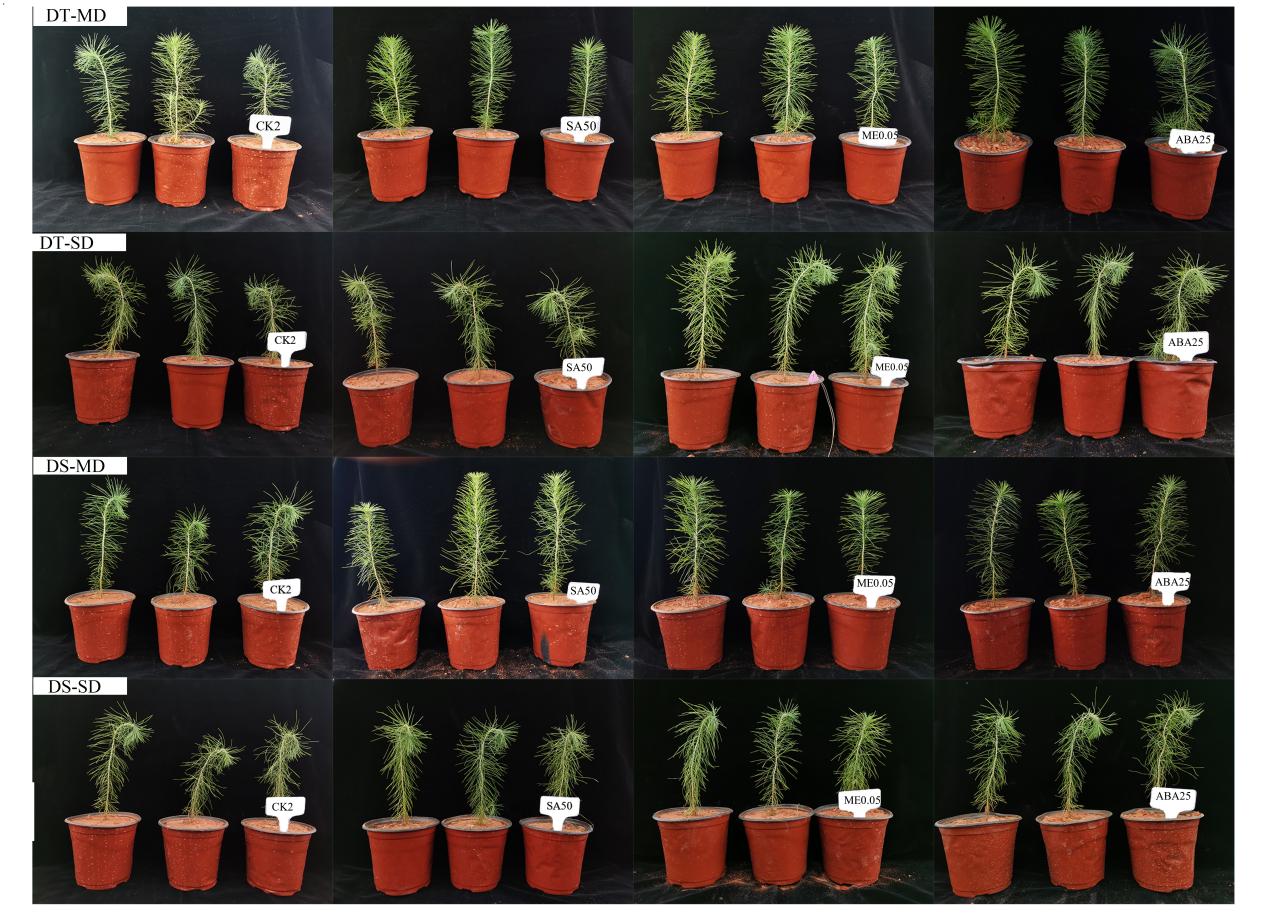


Supplementary Fig 2. The phenotypic changes of *P. massoniana* seedlings treated with different concentrations of exogenous hormones.
