# Supplementary material for: Identification of WRKY transcription factor family genes in Pinus massoniana Lamb. and their expression patterns and functions in response to drought stress
Source: BMC Plant Biol. 2022 Sep 1;22:424. doi: 10.1186/s12870-022-03802-7 (PMC9434871; doi:10.1186/s12870-022-03802-7)
Supplement: Supplementary file 3 — Additional file 3: Supplementary Fig. 3. The schematic diagram of drought stress treatment and sampling process of P. massoniana. [file 12870_2022_3802_MOESM3_ESM.docx]

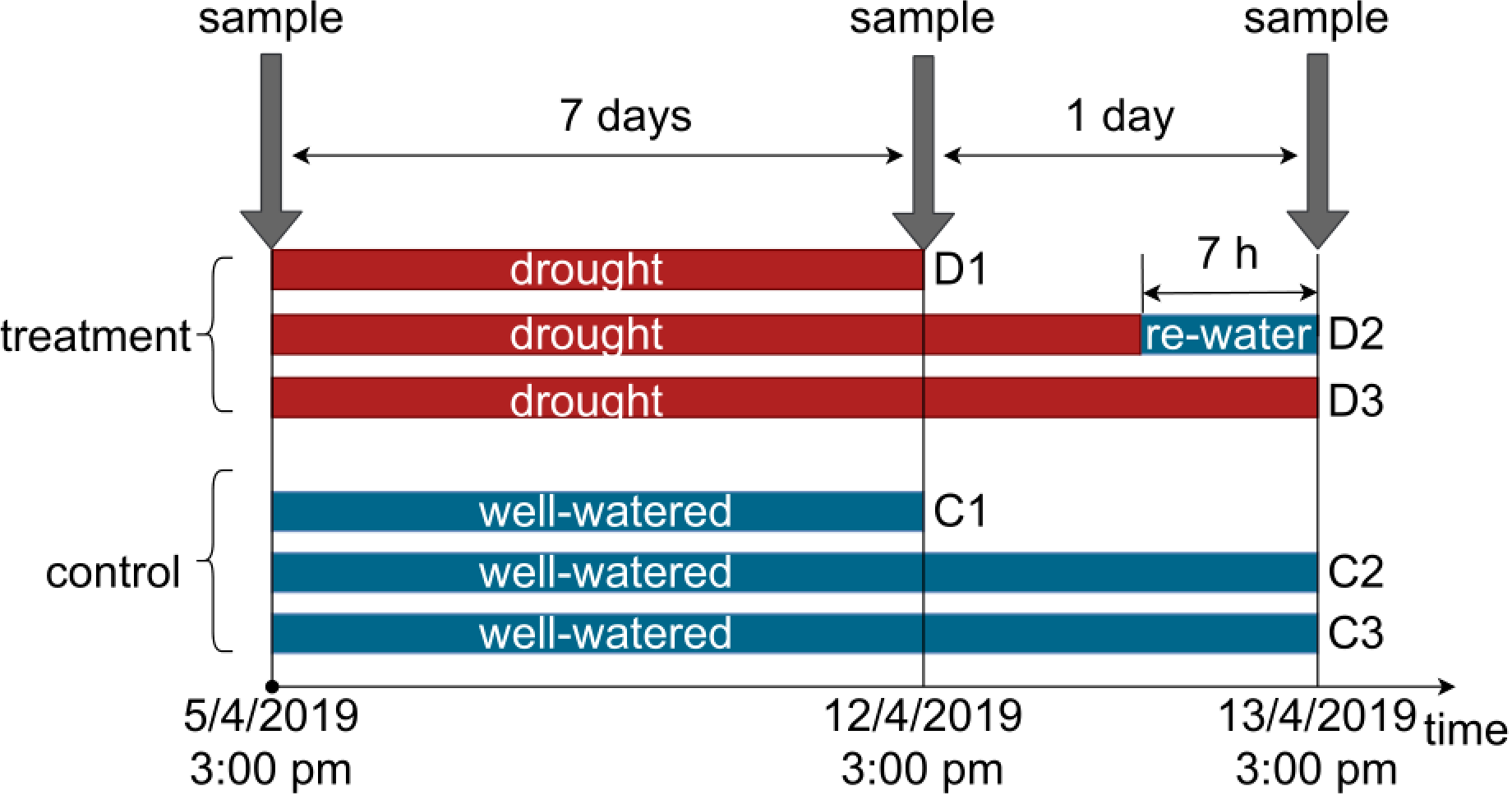


Supplementary Fig 3. The schematic diagram of drought stress treatment and sampling process of *P. massoniana*.
