# Supplementary material for: Identification of WRKY transcription factor family genes in Pinus massoniana Lamb. and their expression patterns and functions in response to drought stress
Source: BMC Plant Biol. 2022 Sep 1;22:424. doi: 10.1186/s12870-022-03802-7 (PMC9434871; doi:10.1186/s12870-022-03802-7)
Supplement: Supplementary file 4 — Additional file 4: Supplementary Table 1. Physical and chemical analysis of WRKY in Pinus massoniana. [file 12870_2022_3802_MOESM4_ESM.docx]

**Supplementary Table 1. Physical and chemical analysis of WRKY in *Pinus massoniana***

| Gene name | Group | Domain | Amino acid number | Isoelectric point | Molecular weigh | Instability index | Hydropathicity | Subcellular localization | Transmembrane structure |
| --- | --- | --- | --- | --- | --- | --- | --- | --- | --- |
| PmWRKY1 | Ⅰ | WRKYGQK×2 | 908 | 6.54 | 98.15 | 55.86 | -0.777 | Nuclear | None |
| PmWRKY2 | Ⅰ | WRKYGQK×2 | 697 | 8.78 | 73.96 | 60.62 | -0.727 | Nuclear | None |
| PmWRKY3 | Ⅰ | WRKYGQK×2 | 656 | 8.93 | 70.81 | 56.20 | -0.797 | Nuclear | None |
| PmWRKY4 | Ⅱb | WRKYGQK | 633 | 6.54 | 69.94 | 44.51 | -0.813 | Nuclear | None |
| PmWRKY5 | Ⅰ | WRKYGQK×2 | 678 | 6.07 | 74.00 | 50.46 | -0.770 | Nuclear | None |
| PmWRKY6 | Ⅲ | WRKYGQK | 477 | 5.56 | 52.48 | 58.87 | -0.689 | Nuclear | None |
| PmWRKY7 | Ⅱc | WRKYGQK | 475 | 5.82 | 50.90 | 73.73 | -0.703 | Nuclear | None |
| PmWRKY8 | Ⅱa | WRKYGQK | 267 | 9.66 | 29.37 | 54.17 | -0.583 | Nuclear | None |
| PmWRKY9 | Ⅱd | WRKYGQK | 342 | 9.56 | 36.92 | 45.40 | -0.678 | Nuclear | None |
| PmWRKY10 | Ⅱe | WRKYGQK | 422 | 4.68 | 45.69 | 61.24 | -0.510 | Nuclear | None |
| PmWRKY11 | Ⅱa | WRKYGQK | 416 | 8.55 | 46.11 | 49.50 | -0.668 | Nuclear | None |
| PmWRKY12 | Ⅱd | WRKYGQK | 348 | 9.42 | 37.52 | 49.81 | -0.510 | Nuclear | None |
| PmWRKY13 | Ⅱa | WRKYGQK | 380 | 8.66 | 42.70 | 50.06 | -0.803 | Nuclear | None |
| PmWRKY14 | Ⅱa | WRKYGQK | 340 | 8.72 | 37.65 | 54.31 | -0.734 | Nuclear | None |
| PmWRKY15 | Ⅱc | WRKYGRK | 249 | 7.61 | 27.91 | 43.53 | -0.786 | Nuclear | None |
| PmWRKY16 | Ⅱe | WRKYGQK | 475 | 5.37 | 50.85 | 51.36 | -0.631 | Nuclear | None |
| PmWRKY17 | Ⅱb | WRKYGQK | 739 | 6.28 | 80.99 | 45.98 | -0.664 | Nuclear | None |
| PmWRKY18 | Ⅱa | WRKYGQK | 387 | 8.87 | 42.87 | 51.34 | -0.687 | Nuclear | None |
| PmWRKY19 | Ⅰ | WRKYGQK×2 | 504 | 9.43 | 54.59 | 54.70 | -0.848 | Nuclear | None |
| PmWRKY20 | Ⅱc | WRKYGKK | 235 | 8.69 | 26.56 | 54.66 | -0.891 | Nuclear | None |
| PmWRKY21 | Ⅰ | WRKYGQK×2 | 734 | 9.08 | 80.25 | 56.38 | -0.716 | Nuclear | None |
| PmWRKY22 | Ⅱd | WRKYGQK | 359 | 9.82 | 40.62 | 67.27 | -0.896 | Nuclear | None |
| PmWRKY23 | Ⅱa | WRKYGQK | 246 | 8.88 | 27.29 | 60.86 | -0.653 | Nuclear | None |
| PmWRKY24 | Ⅱe | WRKYGQK | 443 | 6.47 | 48.05 | 50.31 | -0.677 | Nuclear | None |
| PmWRKY25 | Ⅱd | WRKYGQK | 342 | 9.37 | 38.03 | 50.20 | -0.459 | Nuclear | None |
| PmWRKY26 | Ⅱb | WRKYGQK | 351 | 8.82 | 39.30 | 57.86 | -0.706 | Nuclear | None |
| PmWRKY27 | Ⅱa | WRKYGQK | 274 | 8.44 | 29.87 | 54.73 | -0.608 | Nuclear | None |
| PmWRKY28 | Ⅱd | WRKYGQK | 364 | 9.68 | 40.43 | 61.56 | -0.636 | Nuclear | None |
| PmWRKY29 | Ⅰ | WRKYGQK×2 | 687 | 6.18 | 74.00 | 63.60 | -0.829 | Nuclear | None |
| PmWRKY30 | Ⅱb | WRKYGQK | 642 | 6.06 | 69.36 | 51.87 | -0.618 | Nuclear | None |
| PmWRKY31 | Ⅱc | WRKYGQK | 465 | 4.92 | 51.52 | 63.91 | -0.721 | Nuclear | None |
| PmWRKY32 | Ⅰ | WRKYGQK | 234 | 10.19 | 26.15 | 35.05 | -0.897 | Nuclear | None |
| PmWRKY33 | Ⅱa | WRKYGQK | 400 | 5.88 | 44.30 | 54.86 | -0.737 | Nuclear | None |
| PmWRKY34 | Ⅱc | WRKYGKK | 245 | 8.43 | 28.27 | 56.17 | -0.855 | Nuclear | None |
| PmWRKY35 | Ⅰ | WRKYGQK | 120 | 9.02 | 13.48 | 73.44 | -0.867 | Nuclear | None |
| PmWRKY36 | Ⅰ | WRKYGQK×2 | 531 | 5.72 | 58.10 | 51.08 | -0.697 | Nuclear | None |
| PmWRKY37 | Ⅱd | WRKYGQN | 180 | 9.18 | 19.65 | 41.02 | -0.320 | Nuclear | None |
| PmWRKY38 | Ⅱc | WRKYGQK | 478 | 6.82 | 53.00 | 70.93 | -1.000 | Nuclear | None |
| PmWRKY39 | Ⅰ | WRKYGQK | 177 | 8.97 | 19.61 | 51.31 | -0.307 | Nuclear | None |
| PmWRKY40 | Ⅰ | WRKYGQK | 237 | 10.23 | 27.12 | 36.07 | -0.594 | Nuclear | None |
| PmWRKY41 | Ⅱa | WRKYGQK | 387 | 8.75 | 43.00 | 46.29 | -0.625 | Nuclear | None |
| PmWRKY42 | Ⅱa | WRKYGQK | 396 | 8.00 | 43.45 | 42.25 | -0.533 | Nuclear | None |
| PmWRKY43 | Ⅰ | WTKYGKR | 432 | 8.22 | 48.29 | 46.80 | -0.779 | Nuclear | None |
