# Supplementary material for: Identification of WRKY transcription factor family genes in Pinus massoniana Lamb. and their expression patterns and functions in response to drought stress
Source: BMC Plant Biol. 2022 Sep 1;22:424. doi: 10.1186/s12870-022-03802-7 (PMC9434871; doi:10.1186/s12870-022-03802-7)
Supplement: Supplementary file 5 — Additional file 5: Supplementary Table 2. Effects of exogenous hormones at different concentrations on biomass and root-shoot ratio of Pinus massoniana (19–214 and 19–220) under drought stress. [file 12870_2022_3802_MOESM5_ESM.docx]

**Supplementary Table 2 Effects of exogenous hormones at different concentrations on biomass and root-shoot ratio of *Pinus massoniana* (19–214 and 19–220) under drought stress**

| Treatments | The total biomass | The total aboveground biomass | The total underground biomass | Root-shoot ratio |
| --- | --- | --- | --- | --- |
| **19–214** | | | | |
| CK1 | 1.032±0.148a | 0.928±0.134a | 0.104±0.019b | 0.113±0.014b |
| CK2 | 0.838±0.065b | 0.725±0.075b | 0.113±0.019ab | 0.159±0.037ab |
| SA10 | 0.906±0.233ab | 0.784±0.204b | 0.122±0.037ab | 0.158±0.037ab |
| SA25 | 0.969±0.135ab | 0.843±0.128ab | 0.127±0.035ab | 0.153±0.050b |
| SA50 | 0.897±0.176ab | 0.756±0.191b | 0.141±0.053a | 0.198±0.072a |
| SA100 | 0.868±0.125b | 0.747±0.123b | 0.121±0.040ab | 0.166±0.062ab |
| SA250 | 0.834±0.184b | 0.714±0.153b | 0.120±0.035ab | 0.167±0.025ab |
| CK1 | 1.032±0.148a | 0.928±0.134a | 0.104±0.019b | 0.113±0.014c |
| CK2 | 0.838±0.065b | 0.725±0.075b | 0.113±0.019b | 0.159±0.037b |
| MeJA0.01 | 0.854±0.062b | 0.722±0.054b | 0.132±0.029ab | 0.184±0.043ab |
| MeJA0.1 | 0.852±0.151b | 0.715±0.011b | 0.137±0.047ab | 0.187±0.049ab |
| MeJA0.2 | 0.898±0.164b | 0.750±0.139b | 0.148±0.039a | 0.199±0.048a |
| MeJA0.5 | 0.894±0.182b | 0.743±0.141b | 0.151±0.051a | 0.202±0.047a |
| MeJA1 | 0.895±0.144b | 0.759±0.115b | 0.136±0.037ab | 0.179±0.049ab |
| CK1 | 1.032±0.148a | 0.928±0.134a | 0.104±0.019b | 0.113±0.014b |
| CK2 | 0.838±0.065b | 0.725±0.075b | 0.113±0.019ab | 0.159±0.037a |
| ABA2 | 0.886±0.205b | 0.761±0.186b | 0.125±0.032ab | 0.167±0.040a |
| ABA5 | 0.820±0.080b | 0.707±0.091b | 0.112±0.050ab | 0.164±0.070a |
| ABA10 | 0.932±0.198ab | 0.802±0.173ab | 0.130±0.041ab | 0.165±0.050a |
| ABA25 | 0.954±0.168ab | 0.809±0.144ab | 0.145±0.053a | 0.181±0.059a |
| ABA50 | 0.798±0.136b | 0.690±0.117b | 0.109±0.023b | 0.158±0.022a |
| **19–220** | | | | |
| CK1 | 1.102±0.244a | 0.981±0.224a | 0.121±0.030a | 0.124±0.025b |
| CK2 | 0.867±0.154b | 0.754±0.119b | 0.113±0.040a | 0.147±0.034ab |
| SA10 | 0.862±0.109b | 0.750±0.098b | 0.112±0.021a | 0.150±0.027ab |
| SA25 | 0.959±0.133ab | 0.822±0.098b | 0.137±0.049a | 0.165±0.047a |
| SA50 | 0.932±0.203b | 0.797±0.195b | 0.136±0.024a | 0.178±0.051a |
| SA100 | 0.983±0.150ab | 0.844±0.143ab | 0.139±0.017a | 0.168±0.032a |
| SA250 | 0.876±0.197b | 0.757±0.139b | 0.119±0.070a | 0.152±0.060ab |
| CK1 | 1.102±0.244a | 0.981±0.224ab | 0.121±0.030ab | 0.124±0.025b |
| CK2 | 0.867±0.154b | 0.754±0.119b | 0.113±0.040b | 0.147±0.034b |
| MeJA0.01 | 0.852±0.222b | 0.712±0.165ab | 0.140±0.066ab | 0.191±0.064a |
| MeJA0.1 | 0.786±0.125b | 0.665±0.107ab | 0.122±0.030ab | 0.183±0.035ab |
| MeJA0.2 | 0.908±0.220b | 0.774±0.194ab | 0.133±0.035ab | 0.174±0.032ab |
| MeJA0.5 | 0.975±0.213ab | 0.820±0.193a | 0.155±0.033a | 0.195±0.052a |
| MeJA1 | 0.939±0.149ab | 0.794±0.122ab | 0.145±0.034ab | 0.182±0.028ab |
| CK1 | 1.102±0.244a | 0.981±0.224a | 0.121±0.030ab | 0.124±0.025c |
| CK2 | 0.867±0.154b | 0.754±0.119b | 0.113±0.040b | 0.147±0.034bc |
| ABA2 | 0.999±0.202ab | 0.871±0.164ab | 0.128±0.048ab | 0.145±0.042bc |
| ABA5 | 0.868±0.134b | 0.753±0.122b | 0.116±0.015b | 0.155±0.016b |
| ABA10 | 0.891±0.126b | 0.775±0.117b | 0.116±0.021b | 0.152±0.027bc |
| ABA25 | 0.942±0.152ab | 0.794±0.137b | 0.147±0.026a | 0.188±0.036a |
| ABA50 | 0.962±0.247ab | 0.818±0.211b | 0.144±0.037ab | 0.176±0.011ab |

Note: Different lowercase letters indicate significant differences at the 0.05 level.
