# Supplementary material for: Identification of WRKY transcription factor family genes in Pinus massoniana Lamb. and their expression patterns and functions in response to drought stress
Source: BMC Plant Biol. 2022 Sep 1;22:424. doi: 10.1186/s12870-022-03802-7 (PMC9434871; doi:10.1186/s12870-022-03802-7)
Supplement: Supplementary file 6 — Additional file 6: Supplementary Table 3. qRT-PCR Primer sequences of the genes. [file 12870_2022_3802_MOESM6_ESM.docx]

**Supplementary Table 3 qRT-PCR Primer sequences of the genes**

| Gene name | Forward primer (5′–3′) | Reverse primer (5′–3′) | Length (bp) |
| --- | --- | --- | --- |
| *PmWRKY6* | GGCTGTCAGGCGACGAAGAAAG | GGAGTTGAAGAAGGCGGGCATC | 112 |
| *PmWRKY10* | ACGATGGACCGCCCTCAGATAAG | TCAGTGCAGCTCCGTTCAACTTG | 144 |
| *PmWRKY22* | AATGTAGCAGTGTGAGAGGTTGTCC | GATTCGGGCTTCCAGAGAGTTTCC | 129 |
| *PmWRKY30/31* | ATGATAAGCGATGGGTGCCAATGG | ACCTCTGAACCTGTTTGCGAACG | 127 |
| *PmCYP* | CAAGGGTTCGTCGTTCCAC | GGCAAACTTCTCGCCGTA | 109 |
| *NbAction* | TGGACTCTGGTGATGGTGTCA | ACATGTAACCACGCTCGGTA | 141 |
| *NbAPX* | TTGGCTGGAGTGGTTGCTGTTG | CATCAGGCAGGCGACCTTCAAG | 106 |
| *NbCBL* | GACACGAAGATCCTGTAGTCCTTGC | CCGAACCGCTACTATTCTTGACCAG | 96 |
| *NbCAT* | CCTGTTCGCCATGCTGAGAAGTATC | TTCTTGCCTGTCTGGTGTGAATGAG | 138 |
| *NbWRKY1* | TCACCGCTGGCGCAAATATGG | GCCCTCTCCACATGCTTCTTCAC | 111 |
| *NbWRKY8* | CGACGACGAAGAAGAACCAGACTC | CTGTTCTGCTTCCTGGTGCTGAC | 83 |
| *NbWRKY9* | ACGGCTGCTACTTCACCATCAATG | TTTGCTGGTTGGCTTGTTGTTGC | 94 |
| *NbWRKY10* | TTCACGGGTCAGGGCAGAAGAG | TGCAGGCAGTGTTGGAAGTTGTC | 109 |
| *NbWRKY11* | CCTCCAAGCCCAAGTCCAAGAAC | GCTCCATCAGAATTGCCAACATGC | 146 |
| *NbWRKY12* | GGCGGCAACTGACCCTAAAGC | TTGGCGGTATTGTGGCTACTGTTC | 96 |
| *NbWRKY13* | GGTTTGTTGGGTTCGCCTTTGTTG | CGCTGCTGAAGAAGTCGGATAGTC | 150 |
| *NbWRKY14* | CCTCAACCAAGCAGACGCAGAAG | AAGCAGGGAGAGCACCATTTAAGC | 127 |
| *NbWRKY15* | AATGTTCGTCTCAGGTGGCTTCAG | CTTGTCCGATGGTTCGCTGTCC | 125 |
